# Fusion and fission events regulate endosome maturation and viral escape

**DOI:** 10.1038/s41598-021-86877-w

**Published:** 2021-04-12

**Authors:** Mario Castro, Grant Lythe, Jolanda Smit, Carmen Molina-París

**Affiliations:** 1 Grupo Interdisciplinar de Sistemas Complejos (GISC) and Instituto de Investigación Tecnológica (IIT), Universidad Pontificia Comillas, Madrid, Spain; 2 Department of Applied Mathematics, School of Mathematics, University of Leeds, Leeds, UK; 3 Department of Medical Microbiology and Infection Prevention, University Medical Center Groningen, Groningen, The Netherlands; 4 Theoretical Biology and Biophysics, Theoretical Division, Los Alamos National Laboratory, Los Alamos, NM 87545 USA

**Keywords:** Biological physics, Virus-host interactions

## Abstract

Endosomes are intracellular vesicles that mediate the communication of the cell with its extracellular environment. They are an essential part of the cell’s machinery regulating intracellular trafficking via the endocytic pathway. Many viruses, which in order to replicate require a host cell, attach themselves to the cellular membrane; an event which usually initiates uptake of a viral particle through the endocytic pathway. In this way viruses hijack endosomes for their journey towards intracellular sites of replication and avoid degradation without host detection by escaping the endosomal compartment. Recent experimental techniques have defined the role of endosomal maturation in the ability of enveloped viruses to release their genetic material into the cytoplasm. Endosome maturation depends on a family of small hydrolase enzymes (or GTPases) called Rab proteins, arranged on the cytoplasmic surface of its membrane. Here, we model endosomes as intracellular compartments described by two variables (its levels of active Rab5 and Rab7 proteins) and which can undergo coagulation (or fusion) and fragmentation (or fission). The key element in our approach is the “per-cell endosomal distribution” and its dynamical (Boltzmann) equation. The Boltzmann equation allows us to derive the dynamics of the total number of endosomes in a cell, as well as the mean and the standard deviation of its active Rab5 and Rab7 levels. We compare our mathematical results with experiments of Dengue viral escape from endosomes. The relationship between endosomal active Rab levels and pH suggests a mechanism that can account for the observed variability in viral escape times, which in turn regulate the viability of a viral intracellular infection.

## Introduction

Endosomes are enigmatic organelles that regulate intracellular cargo trafficking^1,2^. These (literally) *inner bodies* are dynamic in movement and have decorated cytoplasmic membranes^3^. They can merge; that is, they can undergo fusion. To avoid confusion, in this manuscript we shall refer to viral RNA release from the endosome to the cytosol as *escape* and to the merging of two endosomes as *fusion*. We note that other authors refer to fusion as the merging of the viral envelope and the endosomal membrane^4^. Endosomes can also break up (fission)^5^ or kiss and run^6^, in a random choreography that allows the cell to sort, recycle or degrade its internalised cargo. This endosomal maturation programme requires a dramatic transformation of these organelles: from early endosomes, to late ones, recycling ones, and degrading lysosomes^1,7^.

In recent years, a lot of effort has been devoted to decipher how viral entry and subsequent intracellular viral genome release are related to endosomal maturation and intracellular trafficking^8^. Current consensus suggests that many viruses, independently of their entry pathway, are delivered to the so-called early endosomes, and thus, enter an intricate network of endosomes: from early to late endosomes, until the endosomal pH is low enough to trigger viral membrane fusion and escape to the cytosol^9,10^. In this way, endosomes become one of the main vehicles for viral intracellular trafficking in the infected cell, transporting viral genomic cargo from the plasma membrane to the cytosol^4^. For many viruses, escape is preceded by a sudden drop in the endosomal pH^8,9,11^. This often occurs in late endosomes, where in fact, high levels of the GTPase Rab7 have been shown to be responsible for viral escape^12,13^. This rather interesting feature seems to indicate that by tracking the active Rab decoration of the endosomal *cytoplasmic surface*, one is in fact, also following the maturation history of individual endosomes. For example, Rab5 is a marker of early endosomes and Rab7 of late ones^14^. Thus, as well as serving as markers of endosome maturation, different Rab proteins have been identified as central regulators of the endosomal transport machinery^15^. Of particular relevance to viral escape, is the fact that the pH of a given endosome has been experimentally linked to its levels of active Rab5 and Rab7 proteins^7,15–17^.

Given the large variability observed in viral escape times^11,13,18^, it is timely to ask ourselves the following questions: (i) is pH alone the main regulator of viral escape events, (ii) is the pH dependence gradual (analogical) or abrupt (digital), (iii) can endosome maturation and its dynamics explain the observed heterogeneity in the distribution of viral escape times, and (iv) what mechanism of endosome maturation is compatible with current empirical observations. Question (i) has already been quantitatively addressed, both experimentally and theoretically^12,19,20^. These studies concluded that a low endosomal pH triggers viral escape. At the biochemical level, a pH drop induces a protein conformational change in the viral envelope protein, which, consequently, drives the fusion between the viral envelope protein and the internal surface of the endosomal membrane. This event initiates the release of viral genetic material into the cytosol, and in turn leads to the virus escaping potential degradation in the lysosome.

In this paper, we aim to provide answers to questions (ii)–(iv) above. We do so by making use of a novel mathematical approach to describe the population dynamics of endosomes containing endocytosed viral particles. The mathematical framework developed and presented here includes the contribution to endosome maturation and dynamics from fusion and fission events, as well as those molecular (biochemical) processes related to active Rab recruitment and endosome acidification. In this way, the method allows one to characterise and quantify the mechanistic details of endosomal maturation with reduced mathematical complexity to enable comparison with experimental data of viral escape.

### The biology of endosomal Rab5 and Rab7 dynamics

**Figure 1:**
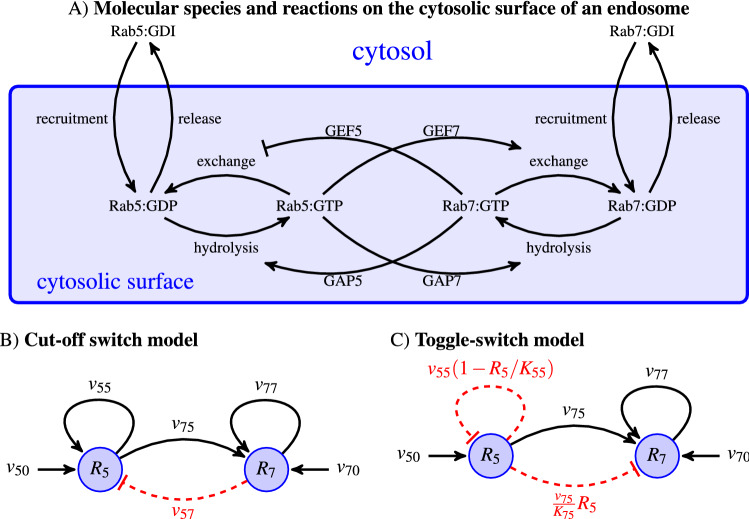
Schematic summary of the biochemical reactions which take place on the cytosolic surface of an endosome and competing hypotheses for Rab5 and Rab7 activation. (**A**) Biochemical reactions that regulate the levels of Rab5 and Rab7 in the cytosol and on the surface of endosomes in inactive (Rab-GDP) and active (Rab-GTP) forms. GDI means GDP-dissociation inhibitor (see Ref.^21,23^ for details). Eqs. (12) and (13) (or Eqs. (14) and (15)) encode the key molecular interactions considered in each mathematical model. (**B**) Cut-off switch model of Rab5 to Rab7 switching. For instance, the red dashed line is modelled by the term proportional to $v_{57} \; x_7$. (**C**) Toggle-switch model of Rab5 to Rab7 switching. The increase of Rab7 with time is caused by a reduction of the activation signal due to an inhibitory one. The level of active Rab5 ($R_5$) decreases with time due to a negative feedback of the active Rab7 ($R_7$). Both models have been adapted from Ref.^21^. The rates $v_{ij}$ will be encoded in the molecular current, ${\vec{{\mathbf{J}}}}$, defined in Eqs. (12) and (13) for the cut-off switch model, and Eqs. (14) and (15) for the toggle-switch model.

Early endosomes (Rab5-positive) undergo a progressive replacement of Rab5 with Rab7^5^. Rabs are not endocytic cargo but endosome surface proteins which can be found in two conformations: Rab5:GDP and Rab5:GTP, inactive and active, respectively. This replacement process involves several molecules and chemical reactions (see a schematic summary in Fig. 1A, adapted from Ref.^21^). Specifically (see Ref.^21^ for details), Rab5-positive endosomes (a signature of early endosomes) gradually become Rab7-positive endosomes (namely, late endosomes)^5^. The complete *endosomal maturation* process then replaces the Rab5 decoration with a Rab7 one. Rab5 and Rab7, guanine nucleotide exchange factors (GEFs), GTPase-activating proteins (GAPs), and effector molecules interact in a series of biochemical reactions (see Fig. 1A) which take place on the endosomal cytosolic surface^22^. The conversion from Rab5 to Rab7 is, in fact, a consequence of programmed and simultaneous changes in the nucleotide cycle of both Rab5 and Rab7, which shuttle between inactive, GDP-bound, and active, GTP-bound, conformations. GEFs catalyse the exchange of GDP into GTP and GAPs catalyse the hydrolysis of GTP into GDP^23,24^. This series of biochemical reactions can be explained by two different and competing hypotheses (see Fig. 1B,C), as comprehensively described in Ref.^21^. The first one is the so-called *toggle-switch model*. It considers a weak repression of Rab7 (by Rab5), which leads to reduced Rab7 activation (see Fig. 1B). The second one, so-called *cut-off switch* model, assumes that Rab7 activation strongly suppresses that of Rab5 (see Fig. 1C). The authors of Ref.^21^ aimed to identify which hypothesis could be best supported by experimental data, and thus, introduced a modular mathematical model (see Appendix C in Ref.^21^). The drawback of the exhaustive model comparison described in Ref.^21^ is the need to make use of specific fitting algorithms to parameterise the models with data from Ref.^25^, as well as its inherent model complexity. We avoid this by clearly encoding the biochemical reactions described in Fig. 1 in the specific choices for the *molecular currents* which will describe active Rab5 and Rab7 endosomal decoration. Once the Rab molecular reactions that take place on the endosomal cytosolic surface are at hand, our framework also considers the interactions of endosomes with other nearby endosomes. In particular, endosomes can undergo fusion and fission^26^, or kiss-and-run^6^ events. The latter process occurs when Rab5 molecules restrict the complete fusion of an endosome, allowing the exchange of solutes between them, but without complete intermixing of their membranes^6^. Here, we shall only consider complete endosomal fusion and fission events.

### Mathematical framework of endosome dynamics and maturation

Mathematical models have helped identify some of the mechanisms involved in the endosomal maturation process. For instance, some regulatory proteins, *e.g.,* SNAREs, Rabs, and other coating proteins, have been linked to the formation, degradation and renewal of endosomes. There is consensus on what mechanisms are involved in these processes, although their individual quantitative contribution has remained elusive^17,27^. In this section, we summarise the biology of endosomal Rab dynamics, as well as previous mathematical modelling efforts.

Most mathematical models of endosome dynamics (with the notable exception of the model in Ref.^28^ which pioneered the approach developed here) make use of a set of ordinary differential equations (ODEs) to describe the dynamics of endosomal Rab protein decoration. This poses a practical problem since, in all cases, the data used to parameterise the mathematical models is averaged over all the endosomes in a cell. However, as we mentioned above, fusion and fission events couple two nearby endosomes, and hence, endosomal averages might not be appropriate to describe the pH of an individual endosome. To illustrate why traditional ODE models do not capture this variability properly, let us consider an extreme case of two endosomes with pH 4 and 7, respectively, and let us assume that viral escape events occur when the endosomal pH is below 5. The average pH will be 5.5 in this case, so viral escape will not occur, which contradicts the fact that it will in one of the endosomes, but not the other (if no average is considered). Thus, endosomal averaging can smooth out fast changes or significant heterogeneities, and in turn lead to the conclusion that viral escape is a continuous process rather than mediated by a pH threshold^27^. At the other end of the modelling spectrum, the biochemistry of proton exchange has been used to describe the thermodynamics of endosome pH acidification^29^. Also, the authors of Ref.^16^ model the acidification of individual endosomes, without considering interactions between them. Finally, it is worth mentioning that, in Ref.^30^ the authors made use of a hybrid method, which includes differential equations for active Rab5, inactive Rab5 and the total number of endosomes. Specifically, the model considers the mean number of Rab5 molecules per endosome and assumes that Rab5 exchange and activation between cytosol and endosomes is proportional to the number of endosomes (see Eqs. (S50)–(S53) in Section 4 of the Supplementary Information, which were proposed by the authors of Ref.^30^). Note that Eq. (S50) incorporates the role of fusion and fission events in terms of the mean number of endosomes. Thus, this *mean-field* approach is unable to describe individual endosome dynamics. While this can be a valuable approximation to characterise endosome population dynamics, it fails to capture endosome heterogeneity, which is essential if we want to understand and describe the observed experimental variability. A suitable method to address this challenge would be the use of agent-based models (ABM)^31^, in which each endosome can be simulated, as an agent, and is described in terms of its molecular content (or cargo). However, ABMs are often computationally expensive or not amenable to parameter inference.

Our approach here takes a different route beyond deterministic descriptions of endosomal dynamics at the cellular level^21,25,30,32^, or at the single endosome level^16,17,27^. As we have discussed earlier, the former presumes that Rab dynamics at the cellular scale can be studied from the observation of endosomal averages, and, the latter, focuses on the biochemical reactions that occur on the cytosolic surface of endosomes, and fails to include the interaction between endosomes. To overcome these limitations, we introduce a model inspired by the dynamics of droplet coalescence, also known in the physics literature as the Smoluchowski equation^28,33,34^. Our approach allows one to recover previous phenomenological models^21,30^ under certain mathematical assumptions (see Section 3 of the Supplementary Information). These models are based on an integro-differential equation which can describe cells^35–37^ or endosomes^28,38^. A difficulty with phenomenological models is their lack of mathematical tractability. To avoid this significant limitation, here, we make use of a formalism that enables one to describe the dynamics of the number of endosomes and their cargo of (active) Rab5 and Rab7 molecules. Furthermore, using the two-dimensional Laplace transform, the formalism naturally leads to analytical expressions for the rate equations (ODEs) of interest. Once the rate equations have been derived, and together with experimental data^10^, we test the validity of these equations and discriminate between two different mechanistic hypotheses^39^. Equipped with this mathematical framework, we can simplify some of the underlying assumptions used in other models (see Section 3 of the Supplementary Information) and perform a model selection analysis (making use of experimental data). Our interest is to directly emphasise the underlying molecular mechanisms rather than certain mathematical choices; this can be done since our approach avoids *ad hoc* reaction terms based on Hill or logistic functions (see the “Results” section).

Finally, and going back to our initial discussion on endosome acidification, we emphasise that endosome acidification is a complex problem and as yet, not fully understood. Traditionally, theories of organelle acidification assumed thermodynamic equilibrium, but cellular compartments are not in equilibrium during endosome maturation. Modern alternatives propose sophisticated dynamical models^29^ to characterise the dynamics of acidification, but they require solving a system of differential equations modelling proton exchange and its interaction with endosomal cations, and involving 22 unknown parameters, which cannot be identified with our experimental data sets. Alternative ways to estimate intracellular pH can be performed with the use of DNA nanomachines^40^ or by luminescence markers of acidity acting as cargo transported by the endosome^41^. Since the experimental error can be as large as $\Delta$pH$\sim 1$, these experiments might not be accurate enough in the case of abrupt pH changes. To overcome these experimental limitations, several approaches have been proposed to understand the interplay between Rab decorations and endosomal pH^16,27^. These approaches, in a nutshell, provide a mechanistic model of proton dynamics and a heuristic interpolation model between early and late endosomes, through an equation of the following kind:

1 $$\begin{aligned} \text{pH}(t)=\text{pH}^\text{early}+(\text{pH}^\text{late}-\text{pH}^\text{early}) \; \frac{\text{Rab}7(t)}{\text{Rab}5(t)+\text{Rab}7(t)}, \end{aligned}$$

where $\text{Rab}5(t)$ and $\text{Rab}7(t)$ have to be understood at the single-endosome level, not at the single-cell level. We shall make use of this equation to derive the time course of endosomal pH, $\text{pH}(t)$, by providing the dynamics of $\text{Rab}5(t)$ and $\text{Rab}7(t)$ as derived from our general mathematical framework.

### Boltzmann equation for the endosomal distribution

In this section, we provide a mathematical description of the biological mechanisms discussed in the previous one. We consider a single cell containing a collection of endosomes. Each endosome is characterised by its active Rab cargo, technically, [Rab5:GTP] and [Rab7:GTP], respectively, $(x_5,x_7)$ at time *t*. The intensity of the fluorescence markers for Rab5 and Rab7 is normalized to the maximum intensity provided by the experimental device. Our variables, $x_5$ and $x_7$ are the normalized intensities and, hence, they are proportional to the number of Rab molecules on the membrane. We introduce

2 $$\begin{aligned} \; n(x_5,x_7;t)\equiv \text { ``per-cell endosomal distribution''}. \end{aligned}$$

Using $x_5, x_7$ as defined above instead of the Rab concentration simplifies the mathematical modelling, since we avoid the need to describe endosomal volumes to compute the concentration. The price paid by these assumptions is that we do not include or consider any such details such as the volume, size or shape of endosomes. Equation (2) allows us to define the observable moments of the distribution defined in the Supplementary Information. For example, the first moments of the distribution have been defined in Eqs. (S1)–(S3).

We can now write the evolution equation for $\; n(x_5,x_7;t)$, that we refer to as the Boltzmann equation for the endosomal distribution. In the following section, we explain the meaning of each term in the equation and its biological rationale.

3 $$\begin{aligned} \frac{\partial \; n(x_5,x_7;t)}{\partial t}&=\underbrace{\frac{1}{2}\int _{0}^{x_5} dx_5'\int _0^{x_7} dx_7'\; K_{FUS}\left( x'_5,x_5-x'_5,x'_7,x_7-x'_7\right) \; n\left( x_5',x_7';t\right) \; n\left( x_5-x'_5,x_7-x'_7;t\right) }_{\text {fusion (gain term)}}\nonumber \\&\underbrace{-\; n(x_5,x_7;t)\int _{0}^{+\infty } dx_5'\int _0^{+\infty } dx_7'\; K_{FUS}\left( x_5,x'_5,x_7,x'_7\right) \; n\left( x_5',x_7';t\right) }_{\text {fusion (loss term)}} \nonumber \\&\underbrace{+\int _{0}^{+\infty } dx_5'\int _{0}^{+\infty } dx_7'\; K_{FIS}\left( x_5,x'_5,x_7,x'_7\right) \; n\left( x_5+x'_5,x_7+x'_7;t\right) }_{\text {fission (gain term)}} \nonumber \\&\underbrace{-\frac{1}{2}\; n(x_5,x_7;t)\int _0^{x_5} dx_5'\int _0^{x_7} dx_7'\; K_{FIS}\left( x'_5,x_5-x'_5,x'_7,x_7-x'_7\right) }_{\text {fission (loss term)}}\nonumber \\&\underbrace{-\mu _0\; n(x_5,x_7;t)}_{\text { degradation}}-\underbrace{\mathbf {\nabla }\cdot {\vec{{\mathbf{J}}}}\left( x_5,x_7\right) }_{\text {Rab5 and Rab7 interactions}}+ \underbrace{S_0 \, \delta (x_5)\, \delta (x_7)}_{\text { endocytosis of viral particle}}. \end{aligned}$$

Although this precise equation has not been proposed before, simplified versions with fewer biological mechanisms^28^ or in fewer dimensions^37,42^ have been studied in different contexts.

### Mathematical modelling hypotheses

**Figure 2:**
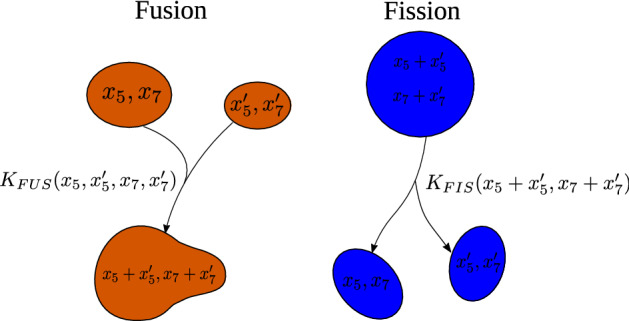
Fusion and fission events depend on the number of Rab5 and Rab7 molecules, not their concentration. Left: two endosomes with different levels of active Rab5 and Rab7, $(x_5,x_7)$ and $(x_5',x_7')$, respectively, merge into a new endosome with levels of active Rab5 and Rab7, $(x_5+x_5',x_7+x_7')$, with rate $K_{FUS}(x_5,x'_5,x_7,x'_7)$. Right: an endosome with levels of active Rab5 and Rab7 $(x_5+x'_5,x_7+x'_7)$ splits into two new endosomes with levels of active Rab5 and Rab7 $(x_5,x_7)$ and $(x_5',x_7')$, respectively, with rate $K_{FIS}(x_5,x'_5,x_7,x'_7)$.

1. *Endocytosis (endosome generation)*: new endosomes are formed by the invagination of the cell membrane. Newly created endosomes (containing endocytosed viral particles) do not have any Rab5 or Rab7 molecules. Progressively, the endosomes become *decorated* with Rab5 and, subsequently, with Rab7 proteins. Mathematically, endosome generation is described by a source term of the form
  4 $$\begin{aligned} S\left( x_5,x_7\right) =S_0 \; \delta \left( x_5\right) \delta \left( x_7\right) \; , \end{aligned}$$
  where $S_0$ is a real positive constant with dimensions of number of endosomes per unit time and the symbol $\delta (\cdot )$ stands for the Dirac delta function.
2. *Endosome degradation/removal*: endosomes can fuse with a lysosome and thus, be removed from the cytosol. In our case, there exists a second form of endosome removal due to viral escape. We assume that the endosomes can be removed at any stage of the maturation process. Hence, we assume a constant removal (or *death*) rate proportional to the number of endosomes, that is independent of its Rab cargo. We write
  5 $$\begin{aligned} \mu \left( x_5,x_7\right) =\mu _0\; n(x_5,x_7;t). \end{aligned}$$
3. *Fusion (coalescence)*: fusion of two endosomes involves the merging of their membranes and, as a consequence, the Rab5 and Rab7 molecules are shared^5^ (see Fig. 2 for a schematic description of this process). In particular, when two endosomes with Rab cargo given by $$(x_5,x_7)$$ and ($$x'_5,x'_7)$$, respectively, fuse, they share their cargo, so the total number of Rab cargo of the newly formed endosome will be $$(x_5+x'_5,x_7+x'_7)$$. The contribution to the time derivative of $$\; n(x_5,x_7;t)$$ is
  6 $$\begin{gathered} \frac{1}{2}\int_{0}^{{x_{5} }} d x_{{5^{\prime}}} \int_{0}^{{x_{7} }} d x_{{7^{\prime}}} K_{{FUS}} \left( {x^{\prime}_{5} ,x_{5} - x^{\prime}_{5} ,x^{\prime}_{7} ,x_{7} - x^{\prime}_{7} } \right)n\left( {x_{{5^{\prime}}} ,x_{{7^{\prime}}} ;t} \right)n\left( {x_{5} - x^{\prime}_{5} ,x_{7} - x^{\prime}_{7} ;t} \right) \hfill \\ - n(x_{5} ,x_{7} ;t)\int_{0}^{{ + \infty }} d x_{{5^{\prime}}} \int_{0}^{{ + \infty }} d x_{{7^{\prime}}} \;K_{{FUS}} \left( {x_{5} ,x^{\prime}_{5} ,x_{7} ,x^{\prime}_{7} } \right)\;n\left( {x_{{5^{\prime}}} ,x_{{7^{\prime}}} ;t} \right), \hfill \\ \end{gathered}$$
  where the first term represents the net *gain* of endosomes with Rab levels $(x_5,x_7)$ and the second one represents the *loss* of endosomes with total levels $(x_5,x_7)$ after fusion with other endosomes. The function $K_{FUS}(x_5,x'_5,x_7,x'_7)$ is referred to as the fusion kernel. It dictates the rate of endosomal fusion, and it clearly depends on the endosomal levels of active Rab molecules.
  A central feature of our model is the consideration of both fusion and fission events. Fusion is enhanced in early endosomes so the rate of fusion correlates positively with the levels of Rab5 and negatively with those of Rab7^5^. For simplicity, we assume that the fusion rate is a linearly increasing function of Rab5 and a linearly decreasing function of Rab7. Hence, we propose
  7 $$\begin{aligned} K_{FUS}\left( x_5,x'_5,x_7,x'_7\right) =K_{FUS}^{(0)}+K_{FUS}^{(5)} \; \left( x_5+x_5'\right) -K_{FUS}^{(7)} \; \left( x_7+x_7'\right), \end{aligned}$$
  where $K_{FUS}^{(k)}$ are constants for $k=0,5,7$. As shown in Ref.^43^, endosomes need to be spatially close to merge. As we are not modelling the intracellular endosome spatial location explicitly, the latter equation favours fusion of early endosomes (higher $x_5$ increases the overall fusion rate) and reduces fusion in late endosomes (it decreases for higher $x_7$). Yet, our model does not preclude fusion events between early and late endosomes^10^; otherwise, our fusion kernel should be multiplied by a Dirac delta function to force fusion only between endosomes with the same levels of $x_5$ and $x_7$.
4. *Fission*: endosomes can divide, thus splitting the amount of Rab5 and Rab7 between the two newly created endosomes. When fission occurs, the total amount of Rab5 and Rab7 is shared randomly between the two newly created endosomes (see Fig. 2 for a schematic description of this process). Similarly to fusion, fission can be described introducing a kernel function, as follows
  8 $$\begin{gathered} \int_{0}^{{ + \infty }} d x_{{5^{\prime}}} \int_{0}^{{ + \infty }} d x_{{7^{\prime}}} K_{{FIS}} \left( {x_{5} ,x^{\prime}_{5} ,x_{7} ,x^{\prime}_{7} } \right)n\left( {x_{5} + x^{\prime}_{5} ,x_{7} + x^{\prime}_{7} ;t} \right) \hfill \\ - \frac{1}{2}n(x_{5} ,x_{7} ;t)\int_{0}^{{x_{5} }} d x_{{5^{\prime}}} \int_{0}^{{x_{7} }} d x_{{7^{\prime}}} K_{{FIS}} \left( {x^{\prime}_{5} ,x_{5} - x^{\prime}_{5} ,x^{\prime}_{7} ,x_{7} - x^{\prime}_{7} } \right). \hfill \\ \end{gathered}$$
  The first term is the *gain* due to the fission of a larger endosome leading to two endosomes, one of them with Rab levels $(x_5,x_7)$. The second one is a *loss* term due to the fission of an endosome with Rab levels $(x_5,x_7)$.
  Endosomal fission is less well understood than fusion. In Ref.^5^ it is suggested that fission occurs randomly at any stage of maturation. Thus, we consider that fission is independent of the number of Rab5 or Rab7 molecules, but that it is not necessarily symmetric (namely, when an endosome splits, the amount of Rab going to each daughter endosome can be different). Mathematically, we propose
  9 $$\begin{aligned} K_{FIS} \left( x_5,x'_5,x_7,x'_7\right) =K_{FIS}^{(0)} \; f\left( |x_5-x'_5|,|x_7-x'_7| \right), \end{aligned}$$
  where, by the symmetric properties of the fission *kernel*^33^, the function *f* satisfies the normalisation condition, $f(0,0)=1$ and is symmetric in its arguments. The simplest case one can consider is symmetric fission; namely, we write
  10 $$\begin{aligned} f\left( x_5-x'_5,x_7-x'_7\right) =\delta \left( x_5-x'_5\right) \; \delta \left( x_7-x'_7\right). \end{aligned}$$
  This choice for *f* means that $50\%$ of each cargo is equally shared between daughter endosomes. Relaxing this assumption only affects the higher moments of the distributions (variance, skewness, etc.) but does not change the mean values of Rab5, Rab7 and the number of endosomes. We discuss this issue in greater detail in the Supplementary Information.
  The contribution to the time derivative of $\; n(x_5,x_7;t)$ is then
  $$\begin{aligned} 4 K_{FIS}^{(0)} \; n\left( 2x_5,2x_7;t\right) - K_{FIS}^{(0)} \; \; n(x_5,x_7;t). \end{aligned}$$
5. *Rab5/Rab7 activation/deactivation*: Rab molecules on the endosomal membrane can be activated after prior incorporation of inactive Rab:GDI from the cytosol. A schematic summary of the reactions of Rab activation/deactivation can be found in Fig. 1.
  In Ref.^21^, the authors considered two competing hypotheses for Rab5/Rab7 activation/deactivation (see Fig. 1B,C). The first one is the *toggle-switch* model and consists in weak repression of Rab7 by Rab5, described by a logistic term. In the second one, the *cut-off switch* model, Rab7 activation strongly suppresses Rab5. To identify which hypothesis was more compatible with the experimental data, the authors introduced a modular model where certain mechanisms could be explained by making use of different mathematical functions (see Supplementary information 1 of Ref.^21^). The drawback of this type of exhaustive model comparison is that specific fitting algorithms had to be adapted to infer model parameters from the data^25^.
  We note that the total number of endosomes does not change with the activation/deactivation of Rab molecules. This fact can be naturally expressed in terms of a conserved quantity. We also note that we model the number of Rab molecules in a given endosome rather than its concentration since the latter one might be affected by fusion and fission events, where the volume or the area of the endosome can significantly change. In the absence of other mechanisms, this conservation law can be expressed in terms of a *molecular current*. The contribution of the dynamics of $x_5$ and $x_7$ for a given endsosome to the time derivative of $\; n(x_5,x_7;t)$ is equal to minus the divergence of a current ${\vec{{\mathbf{J}}}}$:
  $$\begin{aligned} \partial _t\; n(x_5,x_7;t)+\mathbf {\nabla }\cdot {\vec{{\mathbf{J}}}}\left( x_5,x_7\right) =0 \; , \end{aligned}$$
  where
  11 $$\begin{aligned} \mathbf {\nabla }\cdot {\vec{{\mathbf{J}}}}\left( x_5,x_7\right) \equiv \partial _{5} \; J_5\left( x_5,x_7\right) + \partial _{7} \; J_7\left( x_5,x_7\right) \; , \end{aligned}$$
  with $\partial _{k}$ denoting $\partial _{x_k}$, for $k=5,7$. We have also made use of the notation ${\vec{{\mathbf{J}}}}(x_5,x_7)=(J_5(x_5,x_7),J_7(x_5,x_7))$. One must supplement Eq. (11) with *constitutive equations* for the currents, $J_{5}(x_5,x_7)$ and $J_{7}(x_5,x_7)$. In this paper we are going to assume that the currents are proportional to the total number of endosomes, $\; n(x_5,x_7;t)$. This implies that $J_{5}(x_5,x_7)=v_5(x_5,x_7)\; n(x_5,x_7;t)$ and $J_7(x_5,x_7)=v_7(x_5,x_7)\; n(x_5,x_7;t)$, where we have introduced the velocities $v_{5,7}(x_5,x_7)$. The *velocities* $v_{5,7}$ are generic functions of $x_5$ and $x_7$ that need to be prescribed according to the underlying biology of Rab5/Rab7 activation/deactivation discussed earlier. Following Refs.^21,30^, we assume that both molecules evolve and are coupled to each other (see Fig. 1). In other words, the concrete form of the functions $v_{5,7}(x_5,x_7)$ will be determined by performing model selection and thus, identifying the mechanisms that are best supported by the experimental data. Rab5 and Rab7 interact via positive/negative feedback loops. We include these molecular interactions in the current, ${\vec{{\mathbf{J}}}}$, and assume a linear dependence on the number of Rab molecules. For the *cut-off switch* model, one has^21^
  12 $$\begin{aligned} v_5 \left( x_5,x_7\right)&\equiv v_{50}-v_{55}\; x_5-v_{57}\; x_7 \; , \end{aligned}$$
  13 $$\begin{aligned} v_7\left( x_5,x_7\right)&\equiv v_{70}+v_{75}\; x_5-v_{77} \; x_7 \; , \end{aligned}$$
  where the choice of the signs in the coefficients $v_{ij}$ is determined by the network of interactions in Fig. 1. For instance, the inhibition described by the red dashed arrow is captured by the term $-v_{57} \; x_7$. For the *toggle-switch* model one has $v_{57}=0$^21^. Namely, levels of Rab7 do not affect those of Rab5, and thus, the velocity $v_{5}(x_5,x_7)$ does not depend on $x_7$. Yet, the model is non-linear, since it includes two logistic terms. These non-linear terms encode inhibitory mechanisms, as for example, the terms proportional to $v_{55}$ and $v_{75}$. For the *toggle-switch* model one has^21^
  14 $$\begin{aligned} v_5\left( x_5,x_7\right)&\equiv v_{50}-v_{55} \; x_5 \left( 1-\frac{x_5}{K_{55}} \right) \; , \end{aligned}$$
  15 $$\begin{aligned} v_7\left( x_5,x_7\right)&\equiv v_{70}+v_{75} \; x_5 \left( 1-\frac{x_5}{K_{75}} \right) -v_{77} \; x_7 . \end{aligned}$$
  The parameters $K_{55}$ and $K_{75}$ are *carrying capacities* that encapsulate the inhibitory behaviour of Rab5 in the toggle-switch model. As a consequence, we shall show (see Eq. (21)), that the ODE for the endosomal average of Rab5 does not contain the inhibitory feedback proportional to $v_{57}$ present in Eq. (18).

Note that, as newly created endosomes have zero levels of Rab5 and Rab7 (see Eq. (4)), the following boundary conditions for the current ${\vec{{\mathbf{J}}}}$ must be fulfilled:

16 $$\begin{aligned} J_5\left( 0,x_7\right) =0=J_7\left( x_5,0\right). \end{aligned}$$

Intuitively, these equations mean that the Rab5-associated current of endosomes with non-zero levels of Rab7 (first equation) and the Rab7-associated current of endosomes with non-zero levels of Rab5 (second equation) have to be zero.

### Equations for the moments of the Boltzmann distribution

Equation (3) is a non-linear integro-differential equation that is, in principle, analytically intractable. However, it can be simplified under some assumptions that should be carefully scrutinised together with experimental data. In many experimental conditions, only the time evolution of the mean number of molecules of different species (including the total number of endosomes) can be measured. To this end, it is convenient to compute the first moments of the distribution, defined in Eqs. (S1)–(S3).

If we make use of the two-dimensional Laplace transform (for details, see Supplementary Information), we obtain an ODE for the first order moments defined above. For the *cut-off switch* model one can show

17 $$\begin{aligned} \frac{dN(t)}{dt}&= S_0+\left( \frac{K_{FIS}^{(0)}}{8}-\mu _0-K_{FUS}^{(5)} \; R_5(t)+K_{FUS}^{(7)} \; R_7(t)\right) N(t)-\frac{1}{2}K_{FUS}^{(0)}\; N^2(t) \; , \end{aligned}$$

18 $$\begin{aligned} \frac{dR_5(t)}{dt}&= v_{50}\; N(t)-(v_{55}+\mu _0) \; R_5(t) -v_{57}\; R_7(t) \; , \quad \text {and} \end{aligned}$$

19 $$\begin{aligned} \frac{dR_7(t)}{dt}&= v_{70} \; N(t) +v_{75} \; R_5(t)-(v_{77}+\mu _0)\; R_7(t). \end{aligned}$$

For the *toggle-switch* model the ODEs for the first moments can be shown to be

20 $$\begin{aligned} \frac{dN(t)}{dt}&= S_0+\left( \frac{K_{FIS}^{(0)}}{8}-\mu _0-K_{FUS}^{(5)} \; R_5(t)+K_{FUS}^{(7)} \; R_7(t)\right) N(t)-\frac{1}{2}K_{FUS}^{(0)}\; N^2(t) \; , \end{aligned}$$

21 $$\begin{aligned} \frac{dR_5(t)}{dt}&=v_{50} \; N(t)-\mu _0\; R_5 (t) - v_{55} \; R_5(t) \left( 1-\frac{R_5(t)}{K_{55}} \right) \; , \quad \text {and} \end{aligned}$$

22 $$\begin{aligned} \frac{dR_7(t)}{dt}&=v_{70} \; N(t)+ v_{75}\; R_5(t) \left( 1-\frac{R_5(t)}{K_{75}} \right) -(\mu _0+ v_{77})\; R_7(t) \; . \end{aligned}$$

We note that as expected, Eqs. (17) and (20) are the same in both models. We also note that Eq. (3) allows one to derive the dynamical non-linear equation of Refs.^21,30^ (for further details, see Supplementary Information). In all cases, the initial conditions are $R_5(0)=R_7(0)=N(0)=0$.

## Experimental data

**Figure 3:**
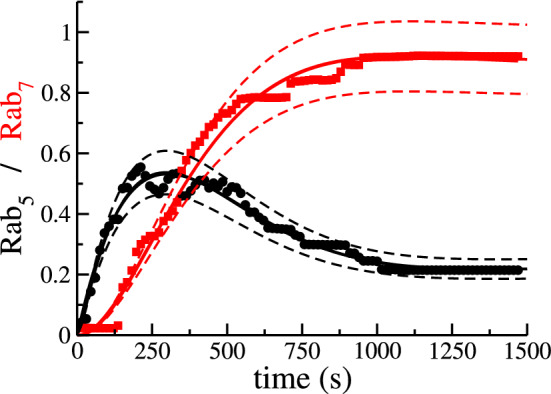
Cut-off switch model captures the mean and fluctuations of the experimental data. Comparison of model predictions (solid lines) as described by Eqs. (17)–(19) and the experimental data (black circles and red squares) from Ref.^10^. Solid lines correspond to the mean values of the normalised intensity of Rab5 and Rab7 fluorescence markers. Dashed lines correspond to the mean ± one standard deviation, as derived from Eqs. (S26)–(S28) in the Supplementary Information making use of the experimentally calibrated model parameters. The fluctuations predicted by the model capture consistently those of the data.

We make use of experimental data obtained by labelling Dengue viral particles (DENV) with the lipophilic fluorescent probe DiD, as previously reported in Ref.^10^ and reproduced in Fig. 3. We use the normalised number of *probes* (DENV viral particles) colocalised with endosomal Rab5 and Rab7 (measured by fluorescence) as an estimate of the number of Rab molecules in endosomes with endocytosed DENV. Here, we are not interested in the DENV lifecycle, but the viral particles will serve as markers to track endosomal dynamics since in the experiments viral particles were colocalised with fluorescent markers for endosomal Rab5 and Rab7 molecules. Overall, 51 escape events were analysed to quantify the levels of Rab5 and Rab7 (see Fig. 3). Analysis of those 51 cases revealed that, although most escape events took place in early endosomes ($86\%$), a non-negligible number of events, $14\%$, took place in Rab5/Rab7-positive intermediate endosomes^10^. In addition, tracking fluorescently labelled endosomes allowed the authors to show that almost half of the endosomes skipped several steps of the maturation process by merging with existing Rab7-positive endosomes (precisely, $45\%$)^10^. Finally, $30\%$ of the tracked endosomes underwent fission events at different stages of their trajectory in the cytoplasm. This supports our choice for a constant fission rate, termed *splitting* in Ref.^10^, which does not depend on the stage of maturation of the endosome, as defined by its Rab cargo $(x_5,x_7)$. Each data point corresponds to a different experiment (until escape occurs), and thus, our model describes the time distribution of the number of endosomes sampled at random times.

## Results

### Data and mathematical modelling best support the cut-off switch hypothesis

**Figure 4:**
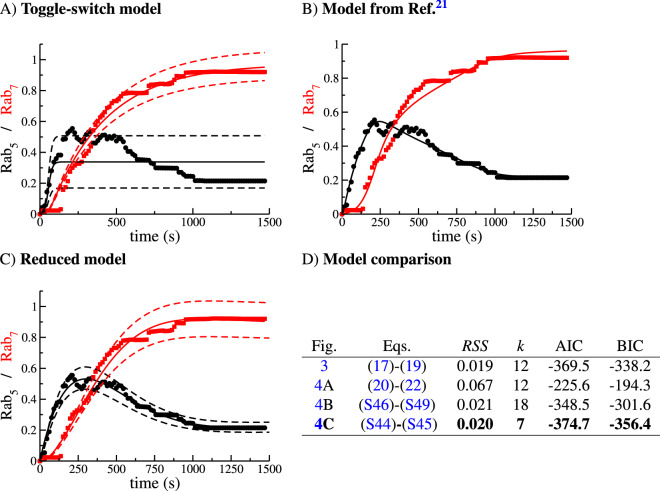
Cut-off switch model (and its reduced version) explains the data more parsimoniously. (**A**) Toggle-switch model, Eqs. (20)–(22). (**B**) Model from Ref.^21^ (see also Eqs. (S46)–(S49) in the Supplementary Information). (**C**) Comparison of the sensitivity-based reduced model, Eqs. (S44)–(S45) (see Supplementary Information for details). (**D**) Model comparison based on the Akaike Information Criterion, Eq. (24) and the Bayesian Information Criterion, Eq. (25). The selected model according to the minimum AIC and BIC is the one shown in boldface. Dashed lines represent the mean ± the standard deviation, as described by Eqs. (S26)–(S28). We note that the formalism introduced here allows one to predict the variance of the estimated solution (**A**,**C**), unlike traditional ODE modelling approaches, where only the mean can be explained (**B**).

Figure 3 shows the experimental data (black circles and red squares) from Ref.^10^ and the result of fitting the data to the mathematical model (cut-off switch) described by Eqs. (17)–(19) (solid lines). A sensitivity analysis of the cut-off switch model (see Table S2 in Supplementary Information) reveals that the most sensitive parameters are, in order of relevance, $K_{FUS}^{(0)}$, $K_{FIS}^{(0)}$, $v_{50}$ and $v_{57}$, and the least relevant, $K_{FUS}^{(7)}$, $K_{FUS}^{(5)}$, $\mu _0$ and $S_0$. The results from our sensitivity analysis are rather interesting since it shows that fusion and fission are essential to understand the experimental data, but that the corrections to the constant term of the fusion kernel, $K^{(0)}_{FUS}$, which are proportional to $K_{FUS}^{(7)}$ and $K_{FUS}^{(5)}$ and depend on the Rab cargo, are negligible. Thus, it seems that a simpler model than the cut-off switch can be used to explain the data, as we show in Section 2 of the Supplementary Information (see also A quasi-linear approximation to describe experimental data section below and Fig. 4C). Furthermore, the source term of new endosomes with zero levels of Rab5 and Rab7 does not significantly affect the dynamics of endosomal Rab5 or Rab7. Numerical integration of Eq. (17) shows that, independently of the initial condition, *N*(0), the number of endosomes quickly approaches a steady state (see Fig. S1). Taking into account the order of magnitude of the parameters in Table S1 of the Supplementary Information and the results of the sensitivity analysis, we find that

23 $$\begin{aligned} \frac{dN(t)}{dt}=&\,S_0+\left( \frac{K_{FIS}^{(0)}}{8}-\mu _0-K_{FUS}^{(5)} \; R_5(t)+K_{FUS}^{(7)} \; R_7(t)\right) N(t)-\frac{1}{2}K_{FUS}^{(0)}\; N^2(t)\simeq 0 \nonumber \\ \Rightarrow&N(t)\simeq N_\text{ss} \equiv \frac{K_{FIS}^{(0)}}{4K_{FUS}^{(0)}} \; , \end{aligned}$$

where we have neglected $K_{FUS}^{(7)}$, $K_{FUS}^{(5)}$, $\mu _0$ and $S_0$ and assumed $N_\text{ss} \ne 0$. As a consequence of *N*(*t*) being almost stationary, the terms $v_{50}N$ and $v_{70}N$ in Eqs. (18) and (19), respectively, are also almost constant. Our theoretical analysis is consistent with our numerical results: Fig. S1 shows that the number of endosomes, *N*(*t*), reaches a value close to its steady state after 40 s. Given the best-fit parameters from Table S1 in the Supplementary Information, we conclude that fusion and fission events are more relevant for the dynamics of Rab5 and Rab7 molecules than the rate of endosome generation since we have $S_0\ll K_{FUS}^{(0)}N^2$ and $S_0\ll K_{FIS}^{(0)}N$. Finally, we now explore the role of the parameter $v_{57}$ in the decrease of $R_5(t)$ at the time of increase of $R_7(t)$. This coefficient encodes the inhibitory effect of Rab7 on the dynamics of Rab5. On the one hand, the sensitivity analysis of the cut-off switch model (see Table S2 in the Supplementary Information) gives this parameter a normalised value of 0.7543 and on the other hand, the best-fit parameters from Table S1 in the Supplementary Information, suggest a value of $4 \times 10^{-3}$ s$^{-1}$ for $v_{57}$. These two results together show the qualitative and quantitative importance of this parameter in the cut-off switch model, which in turn, and in light of the experimental data, supports the cut-off switch hypothesis, in agreement with Ref.^21^. Note that $v_{57}=0$ in the toggle-switch hypothesis, since Rab7 does not affect the dynamics of Rab5. To further test this conclusion, we have also fitted the toggle-switch model, Eqs. (20), (21), (22), to the experimental data. The results are shown in Fig. 4A. It can be concluded that the toggle-switch model cannot explain the decrease of Rab5 observed in the data. We first note that in Eq. (21) $R_5(t)$ does not depend on $R_7(t)$. We then argue that for this model, only fine-tuned mathematical functions of $R_5$ might explain the decrease of $R_5$ at late times. Yet, there is biological evidence to support that the Rab5 decrease and the Rab7 decrease are not independent events. Possibly, more sophisticated mathematical models, as those explored in Ref.^21^, might provide better agreement with the experimental data. Still, simply adding more mathematical terms (and so more parameters) to the dynamical equations would obscure our ability to systematically select between plausible biological mechanisms regulating the dynamics of Rab5 and Rab7.

### A quasi-linear approximation to describe experimental data

Ziegler et al. in Ref.^30^ considered Rab5-dependent fusion and fission of endosomes, making use of a differential equation similar to Eq. (17). Del Conte-Zerial et al. in Ref.^21^ modelled the conversion of early endosomes into late endosomes, which assumes the dynamical replacement of Rab5 by Rab7 during endosome maturation. Their equations, summarised in the Supplementary Information, require a larger number of parameters than those in the cut-off switch model, Eqs. (17)–(19), but do not include a dynamical equation for the number of endosomes. In Fig. 4B we show the fit of their model to the DENV data. Comparison of Figs. 3 and 4 shows that a model that includes the dynamics of the number of endosomes can explain the molecular mechanisms parsimoniously. Our model does not explicitly consider inactive Rabs ([Rab5-GDP] and [Rab7-GDP]) because their levels remain almost constant (see Supplementary information of Ref.^21^). On the other hand, our framework provides dynamical equations for the variance of the number of molecules, as shown in Fig. 4A,C.

In order to perform quantitative model selection, we show in Fig. 4D the value of the Akaike Information Criterion (AIC) for the three models, defined as:

24 $$\begin{aligned} \text {AIC}\equiv n\log \left( RSS\right) +2k \; , \end{aligned}$$

where RSS is the residual sum of squares, *n* the number of points in the data series, and *k* the number of parameters in the mathematical model. Similarly, we compute the Bayesian Information Criterion (BIC), defined as

25 $$\begin{aligned} \text {BIC}\equiv n\log \left( RSS\right) +k\log n \; . \end{aligned}$$

Both methods quantify the goodness of fit but introduce a *penalty* on the number of parameters (the lower the better). As the RSS is similar in all the models (and $n=100$ is the same for all of them), the most decisive factor is the number of parameters, *k*, thus, pointing at the reduced model, given by Eqs. (S44) and (S45) (see Fig. 4C). Thus, we conclude that a mathematical model that considers the mean number of endosomes in a quasi-steady state, $N_\text{ss} \equiv \frac{K_{FIS}^{(0)}}{4K_{FUS}^{(0)}}$, and assumes linear dynamics for the evolution of Rab5 and Rab7 (see Eqs. (S44) and (S45)) is the best candidate for future model extensions. Clearly, the toggle-switch model cannot capture the behaviour of the experimental data, as argued in the previous section. This emphasises an important conclusion that can be derived from our analysis: fusion, fission and Rab7 inhibition of Rab5 are the main mechanisms regulating endosome maturation and, in our context, the endosome acidification which drives viral escape.

### Fluctuations explain the variability of pH-driven viral escape

**Figure 5:**
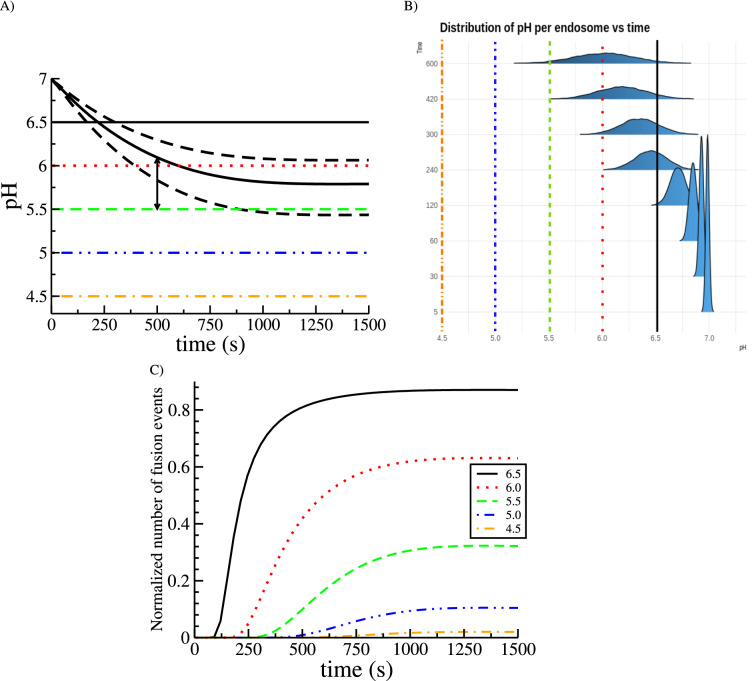
Cut-off switch model predicts large endosomal pH fluctuations. (**A**) Time evolution of the mean endosomal pH (thick curved line) computed from Eqs. (17)–(19) and Eq. (1), and mean ± standard deviation (computed from Eqs. (S26)–(S28) in Supplementary Information, curved dashed lines). The horizontal colour lines correspond to different pH thresholds that could regulate and trigger virus endosomal escape. (**B**) Similar to (**A**) but showing the sampled distribution of pH using Eq. (26). Note how the distribution broadens with time, thus increasing the probability of crossing a given pH threshold. (**C**) Normalised number of viral escape events quantified as the probability of pH being below a threshold (see colour-coded legend), making use of Eq. (26), namely, $\mathbb {P}[{\mathcal N}({\overline{{\text{pH}}}},\sigma _{\text{pH}})<\textrm{threshold}]$.

There exists a simple connection between the levels of endosomal Rab5, Rab7 and endosomal pH given by Eq. (1) (for details, please, see Ref.^16^). In our case, the estimated time evolution of pH is given in Fig. 5A, where we have used the best-fit parameters from Fig. 3 (see Table S1 in the Supplementary Information). The shape of the curve is consistent with previous results^16,29,44^. Since we can compute the second-order moments of the distribution, we can evaluate the standard deviation of the pH (dashed lines in Fig. 5A).

As we mentioned in the Introduction, many intracellular processes are initiated by low (below threshold) values of the pH^10–12^. Yet, as shown in Fig. 5A, the pH fluctuates due to the rich dynamics of endosome maturation and Rab decoration. This variability can only be accounted for if one considers the collective dynamics of the population of endosomes, as described by the distribution $\; n(x_5,x_7;t)$.

The exact distribution for the number of endosomes with a given pH cannot be computed analytically, as it would involve: (i) computing the inverse Laplace transform of $\; \hat{n}(z_5,z_7;t)$, and (ii) computing the marginal distribution

$$\begin{aligned} n(\text {pH})=\iint dx_5 \; dx_7 \;n(x_5,x_7;t) \; \delta \left[ \text {pH}-\left( \text{pH}^\text{early}+\left( \text{pH}^\text{late}-\text{pH}^\text{early}\right) \; \frac{x_7}{x_5+x_7}\right) \right] \; . \end{aligned}$$

Instead, we can exploit this variability to approximate the fluctuations by a normal distribution (Central Limit Theorem). In particular, we can write

26 $$\begin{aligned} {\text{pH}}(t)\sim {\mathcal N}\left[ {\overline{{\text{pH}}}}(t),\sigma _{\text{pH}}(t)\right] \; , \end{aligned}$$

where $\sim \mathcal N$ denotes normally distributed, and ${\overline{{\text{pH}}}}$ and $\sigma _{\text{pH}}$ are the mean and standard deviation of the pH (solid and dashed lines in Fig. 5A, respectively). In Fig. 5B we show synthetically generated histograms according to Eq. (26) to emphasise the role of the width of the distribution and how it affects the probability of having a pH below a certain threshold. The resulting probabilities are shown in Fig. 5C for different values of the pH threshold. For instance, the green dashed line in Fig. 5C shows that at time 500 s, the probability of finding endosomes with pH below 5.5 is already $10\%$, although the mean pH is above 6.0 at that time (see the arrows in Fig. 5). This probability represents also the normalised number of viral escape events (or fraction of *successful* events); that is, for a virus and a fiducial (fixed but arbitrary) pH escape threshold, it gives the probability of viral escape. For instance, under ideal conditions, a virus that requires a pH=4.5 to escape would only have a maximal probability of success of 0.022 ($\sim 2\%$). This, in addition to the large mean time to achieve that probability (that would allow the endosome to fuse with the lysosome and, thus, destroy the intracellular virus), would make the infection non-viable.

This result is of great relevance in the understanding of endosomal viral escape events. For instance, in Ref.^10^ (whose data we are using in the present work), analysis of the experiments revealed that escape events occurred from 300 s post-entry (viral endocytosis). Moreover, colocalisation of escape events with levels of Rab5 and Rab7 also showed that around $5\%$ of the viral particles escaped from within Rab5-positive early endosomes (with no active Rab7 molecules). This implies that the quick pH drop after those early endosomes merged with more acidic endosomes is ultimately regulating the initiation of viral escape events.

## Discussion and conclusions

Traditional mathematical methods, based on ordinary differential equations, can only tell a part of the story when describing systems with a small number of “particles”, such as endosomes in the present case, and where heterogeneity can be large. Deterministic approaches can be a good approximation if one is interested in average numbers or trends, but exclusively at the cell population level. However, in those cases where the response of a system to small variations of a parameter is abrupt (as in the case of viral escape to a pH threshold), averaging can provide confounding answers or lead to mathematical models requiring a large number of parameters. One of the main results of the present work is that, while detailed models of Rab5 and Rab7 dynamics can be found to fit accurately to experiments, such as in Refs.^21,30^, a description based on individual endosomes, decorated with Rab molecules (defined by the distribution $\; n(x_5,x_7;t)$), and their interactions (characterised by fusion and fission events), provides a natural link to the underlying biological mechanisms. The mathematical framework proposed here also allows us to characterise the fluctuations beyond the mean number of endosomes or Rab molecules, since higher-order moments can be computed from the endosomal distribution $\; n(x_5,x_7;t)$. As such, we can determine the time course of the variance in the number of Rab molecules and the covariance.

We have shown that fusion and fission events regulate the maturation and dynamics of endosomes. Even when we have only considered linear constitutive equations, such as $K_{FUS}(x_5,x'_5,x_7,x'_7)$ or ${\vec{{\mathbf{J}}}} (x_5,x_7)$, they have allowed us to capture the complexity of the problem at hand. For instance, more detailed models where kiss-and-run^6^ or vesicle budding events ^45^, decoupled from the rates of fusion/fission, might help to quantify the relative role of those mechanisms. Our approach also sheds some light on the variability of other processes relying on this maturation. As an illustration, as it has been shown for many different viruses^10–12^, viral escape times (after pH drop) are rather heterogeneous, even though endosomal acidification takes place in a gradual manner. For instance, in the case of DENV^10^, it was already reported that some viral particles escaped as early as a few seconds after entry via endocytosis and that, in those cases, fusion with a more acidic endosome preceded that escape event. So, understanding endosome dynamics can be relevant to ascertain the role of different entry pathways in the subsequent fate of viral particles, since different receptors deliver the virus into distinct populations of early endosomes^46^.

Finally, and from a pragmatic perspective, while Eq. (3) has proven useful to study the time evolution of the number of endosomes, and the total number of active Rab5 and Rab7 molecules on the endosomal membrane, it is still a complex system of ODEs, hard to solve analytically. Thus, computational methods aimed to find solutions to these equations can generate rather valuable information. For example, knowledge of the exact distribution of endosomes and their Rab5 and Rab7 cargo, $\; n(x_5,x_7;t)$, would provide the cell *endosomal pH spectrum*, namely, the number of endosomes with a certain pH, $n(\text {pH},t)$, that could be compared with recent experimental methods aimed to quantify intracellular pH^40^. Also, this formalism can be translated to other contexts or scales: *n*(*x*, *y*; *t*) might be seen as the number of cells with a certain expression level of receptors *x* and *y*. Thus, solving the corresponding Smoluchowski equation for *n*(*x*, *y*; *t*) would be a theoretical metaphor of flow cytometry experiments. Another interesting and timely application of our mathematical framework is that of mitochondrial dynamics and interactions. In this case, and in analogy with the endosomes, mitochondria are subject to fusion and fission events modulated by different cargo species (*e.g.,* Ca$^{2+}$, ATP, reactive oxygen species, mtDNA, etc.)^47^. These applications will be considered in future work and are out of the scope of this manuscript.

## Supplementary Information

Supplementary Information.
