# Supplementary material for: Fusion and fission events regulate endosome maturation and viral escape
Source: Sci Rep. 2021 Apr 12;11:7845. doi: 10.1038/s41598-021-86877-w (PMC8041880; doi:10.1038/s41598-021-86877-w)
Supplement: Supplementary file 1 — Supplementary Information. [file 41598_2021_86877_MOESM1_ESM.pdf]

Supporting Information: Fusion and fission events regulate endosome maturation and viral escape

Mario Castro [1,+,*], Grant Lythe [2], Jolanda Smit [3], and Carmen Molina-París [2,4,+,*]

[1] Universidad Pontificia Comillas, Grupo Interdisciplinar de Sistemas Complejos (GISC) and DNL, Madrid, Spain.
[2] Department of Applied Mathematics, School of Mathematics, University of Leeds, Leeds, UK.
[3] Department of Medical Microbiology and Infection Prevention, University Medical Center Groningen, Groningen, The Netherlands.
[4] Theoretical Biology and Biophysics, Theoretical Division, Los Alamos National Laboratory, Los Alamos, NM 87545, USA.
[*] marioc@comillas.edu, molina-paris@lanl.gov
[+]These authors contributed equally.

Section 1: Moments of the endosome distribution

The first-order moments of the distribution $n(x_5,x_7;t)$ are

$$N(t) \equiv \int_0^{+\infty} dx_5 \int_0^{+\infty} dx_7 \; n(x_5,x_7;t) \quad \text{(total number of endosomes)}\,, \tag{S1}$$

$$R_5(t) \equiv \int_0^{+\infty} dx_5\, x_5 \int_0^{+\infty} dx_7 \; n(x_5,x_7;t) \quad \text{(total cargo of Rab5:GTP)}\,, \quad \text{and} \tag{S2}$$

$$R_7(t) \equiv \int_0^{+\infty} dx_5 \int_0^{+\infty} dx_7\, x_7 \; n(x_5,x_7;t) \quad \text{(total cargo of Rab7:GTP)}\,. \tag{S3}$$

We can also define second order moments of the distribution as follows

$$\sigma_5^2(t) \equiv \int_0^{+\infty} dx_5\, x_5^2 \int_0^{+\infty} dx_7 \; n(x_5,x_7;t) - R_5^2(t) \quad \text{(variance of } R_5\text{)}\,, \tag{S4}$$

$$\sigma_7^2(t) \equiv \int_0^{+\infty} dx_5 \int_0^{+\infty} dx_7\, x_7^2 \; n(x_5,x_7;t) - R_7^2(t) \quad \text{(variance of } R_7\text{)}\,, \quad \text{and} \tag{S5}$$

$$\sigma_{57}(t) \equiv \int_0^{+\infty} dx_5\, x_5 \int_0^{+\infty} dx_7\, x_7 \; n(x_5,x_7;t) - R_5(t)R_7(t) \quad \text{(covariance between } R_5 \text{ and } R_7\text{)}\,. \tag{S6}$$

Section 2: Two-dimensional Laplace transform and moment equations

In analogy with the Laplace transform in one dimension, we introduce the two-dimensional Laplace transform, as follows

$$\mathscr{L}[\,n(x_5,x_7;t)] \equiv \int_{0^-}^{+\infty} dx_5 \int_{0^-}^{+\infty} dx_7\; e^{-z_5 x_5 - z_7 x_7}\, n(x_5,x_7;t)\,, \tag{S7}$$

which is a function of the variables z_5 and z_7, and time t. We introduce the following notation for the two-dimensional Laplace transform

$$\hat{n}(z_5,z_7;t) \equiv \mathscr{L}[\,n(x_5,x_7;t)]\,. \tag{S8}$$

We can also define *partial* (or one-dimensional) Laplace transforms associated with each variable, x_5 and x_7, as follows

$$\mathscr{L}_5[\,n(x_5,x_7;t)] \equiv \int_{0^-}^{+\infty} dx_5\; e^{-z_5 x_5}\, n(x_5,x_7;t)\,, \quad \mathscr{L}_7[\,n(x_5,x_7;t)] \equiv \int_{0^-}^{+\infty} dx_7\; e^{-z_7 x_7}\, n(x_5,x_7;t)\,. \tag{S9}$$

Eq. (S7) allows one to derive useful expressions to rewrite the Boltzmann equation, Eq. (5) in the main text, in terms of the Laplace transform of $n(x_5,x_7;t)$. Thus, if we make use of the notation introduced in Eq. (S8), it can be shown that the following

expressions hold[48]:

$$\mathscr{L}[x_5\, n(x_5,x_7;t)] = -\partial_{z_5}\, \hat{n}(z_5,z_7;t)\ , \tag{S10}$$
$$\mathscr{L}[x_7\, n(x_5,x_7;t)] = -\partial_{z_7}\, \hat{n}(z_5,z_7;t)\ , \tag{S11}$$
$$\mathscr{L}[\, n(x_5,x_7;t) * n(x_5,x_7;t)] = [\hat{n}(z_5,z_7;t)]^2\ , \tag{S12}$$
$$\mathscr{L}[x_5\, n(x_5,x_7;t) * n(x_5,x_7;t)] = -\partial_{z_5}\, [\hat{n}(z_5,z_7;t)]^2\ , \tag{S13}$$
$$\mathscr{L}[x_7\, n(x_5,x_7;t) * n(x_5,x_7;t)] = -\partial_{z_7}\, [\hat{n}(z_5,z_7;t)]^2\ , \tag{S14}$$
$$\mathscr{L}[n(2x_5,2x_7;t)] = \frac{1}{4}\, \hat{n}(z_5/2,z_7/2;t)\ , \tag{S15}$$
$$-\mathscr{L}[\partial_{x_5} J_5(x_5,x_7)] = -z_5 \hat{J}_5(z_5,z_7) + \mathscr{L}_7[J_5(0,x_7)]\ , \tag{S16}$$
$$-\mathscr{L}[\partial_{x_7} J_7(x_5,x_7)] = -z_7 \hat{J}_7(z_5,z_7) + \mathscr{L}_5[J_7(x_5,0)]\ , \tag{S17}$$
$$\mathscr{L}[\delta(x_5)\delta(x_7)] = 1\ , \tag{S18}$$

where $\hat{J}_k(x_5,x_7)$ is the Laplace transform of J_k (for $k=5,7$) and the symbol "$*$" denotes the convolution; that is,

$$n(x_5,x_7;t) * n(x_5,x_7;t) \equiv \int_0^{x_5} dx_5' \int_0^{x_7} dx_7'\, n(x_5',x_7';t)\, n(x_5-x_5',x_7-x_7';t)\ .$$

We note that the boundary conditions (see Eq. (16) in the main text) imply

$$\mathscr{L}_7[J_5(0,x_7)] = 0 = \mathscr{L}_5[J_7(x_5,0)]\ . \tag{S19}$$

We now make use of the above properties to compute the Laplace transform of Eq. (5) in the main text. We do so, term by term, as follows:

- **Time derivative:** as the Laplace transform does not involve the time variable, t, we have:

$$\partial_t\, n(x_5,x_7;t) \xrightarrow{\mathscr{L}} \partial_t\, \hat{n}(z_5,z_7;t)\ .$$

- **Fusion (1):** we make use of Eq. (S12),

$$\frac{1}{2}\int_0^{x_5} dx_5' \int_0^{x_7} dx_7'\, K_{FUS}^{(0)}\, n(x_5',x_7';t) n(x_5-x_5',x_7-x_7';t) \xrightarrow{\mathscr{L}} \frac{K_{FUS}^{(0)}}{2}\, [\hat{n}(z_5,z_7;t)]^2\ ,$$

  since $K_{FUS}(x_5',x_5-x_5',x_7',x_7-x_7') = K_{FUS}^{(0)} + K_{FUS}^{(5)}\, x_5 - K_{FUS}^{(7)}\, x_7$.

- **Fusion (2):** we make use of Eq. (S13)

$$\frac{1}{2}\int_0^{x_5} dx_5' \int_0^{x_7} dx_7'\, K_{FUS}^{(5)}\, x_5\, n(x_5',x_7';t) n(x_5-x_5',x_7-x_7';t) \xrightarrow{\mathscr{L}} -\frac{K_{FUS}^{(5)}}{2} \partial_{z_5}\, [\hat{n}(z_5,z_7;t)]^2\ .$$

- **Fusion (3):** we make use of Eq. (S14)

$$-\frac{1}{2}\int_0^{x_5} dx_5' \int_0^{x_7} dx_7'\, K_{FUS}^{(7)}\, x_7\, n(x_5',x_7';t) n(x_5-x_5',x_7-x_7';t) \xrightarrow{\mathscr{L}} \frac{K_{FUS}^{(7)}}{2} \partial_{z_7}\, [\hat{n}(z_5,z_7;t)]^2\ .$$

- **Fusion (4):** we make use of the definition of $N(t)$ in Eq. (S1)

$$-\, n(x_5,x_7;t)\int_0^{+\infty} dx_5' \int_0^{+\infty} dx_7'\, K_{FUS}^{(0)}\, n(x_5',x_7';t) = -K_{FUS}^{(0)} N(t)\, n(x_5,x_7;t) \xrightarrow{\mathscr{L}} -K_{FUS}^{(0)} N(t)\, \hat{n}(z_5,z_7;t)\ .$$

- **Fusion (5):** we make use of Eq. (S10) and the definitions of $N(t)$ and $R_5(t)$ in Eq. (S1) and Eq. (7) in the main text, respectively

$$-\, n(x_5,x_7;t)\int_0^{+\infty} dx_5' \int_0^{+\infty} dx_7'\, K_{FUS}^{(5)}\, (x_5+x_5')\, n(x_5',x_7';t) = -K_{FUS}^{(5)} [x_5 N(t)\, n(x_5,x_7;t) + R_5(t)\, n(x_5,x_7;t)]$$

$$\xrightarrow{\mathscr{L}} K_{FUS}^{(5)} [N(t)\partial_{z_5}[\, \hat{n}(z_5,z_7;t)] - R_5(t)\, \hat{n}(z_5,z_7;t)]\ .$$

- **Fusion (6):** we make use of Eq. (S11) and the definitions of $N(t)$ and $R_7(t)$ in Eq. (S1) and Eq. (S3), respectively

$$n(x_5,x_7;t)\int_0^{+\infty}dx_5'\int_0^{+\infty}dx_7'\,K_{FUS}^{(7)}\,(x_7+x_7')\,n(x_5',x_7';t)=K_{FUS}^{(7)}\,[x_7N(t)\,n(x_5,x_7;t)+R_7(t)\,n(x_5,x_7;t)]$$

$$\xrightarrow{\mathscr{L}}K_{FUS}^{(7)}\,[-N(t)\partial_{z_7}[\,\hat{n}(z_5,z_7;t)]+R_7(t)\,\hat{n}(z_5,z_7;t)]\ .$$

- **Fission (1):** we make use of Eq. (8) in the main text and Eq. (S15)

$$\int_0^{+\infty}dx_5'\int_0^{+\infty}dx_7'\,K_{FIS}^{(0)}\,\delta(x_5-x_5')\delta(x_7-x_7')n(x_5+x_5',x_7+x_7';t)=K_{FIS}^{(0)}\,n(2x_5,2x_7;t)\xrightarrow{\mathscr{L}}\frac{K_{FIS}^{(0)}}{4}\,\hat{n}(z_5/2,z_7/2;t)\ .$$

- **Fission (2):** we make use of Eq. (8) in the main text

$$-\frac{1}{2}\,n(x_5,x_7;t)\int_0^{x_5}dx_5'\int_0^{x_7}dx_7'\,K_{FIS}(x_5',x_5-x_5',x_7',x_7-x_7')$$

$$=-\frac{1}{2}\,n(x_5,x_7;t)\int_0^{x_5}dx_5'\int_0^{x_7}dx_7'\,K_{FIS}^{(0)}\,\delta(x_5'-(x_5-x_5'))\delta(x_7'-(x_7-x_7'))\xrightarrow{\mathscr{L}}-\frac{K_{FIS}^{(0)}}{8}\,\hat{n}(z_5,z_7;t)\ .$$

- **Fission (2'):** Alternative choices of the function f in Eq. (8) in the main text can be used to include asymmetric division. Thus, we would replace the last two terms by

$$\frac{K_{FIS}^{(0)}}{4}\hat{f}(z_5/2,z_7/2)\,\hat{n}(z_5/2,z_7/2;t)-\frac{K_{FIS}^{(0)}}{8}\hat{f}(z_5,z_7)\,\hat{n}(z_5,z_7;t)\ .$$

We note that considering an asymmetric fission kernel will only modify the second and higher order moments of the distribution, but not its first order moments (means). This is due to the symmetries $x_5\leftrightarrow x_5'/x_7\leftrightarrow x_7'$, so that f satisfies

$$\partial_{x_5}f(x_5,x_7)|_{x_5=x_7=0}=0=\partial_{x_7}f(x_5,x_7)|_{x_5=x_7=0}\ .$$

- **Degradation:** we make use of Eq. (5) in the main text and Eq. (S7)

$$-\mu_0\,n(x_5,x_7;t)\xrightarrow{\mathscr{L}}-\mu_0\,\hat{n}(z_5,z_7;t)\ .$$

- **Divergence of the current (1):** we make use of Eq. (S16)) and Eq. (S19)

$$-\partial_{x_5}J_5(x_5,x_7)\xrightarrow{\mathscr{L}}-z_5\hat{J}_5(z_5,z_7)\ .$$

- **Divergence of the current (2):** we make use of Eq. (S17)) and Eq. (S19)

$$-\partial_{x_7}J_7(x_5,x_7)\xrightarrow{\mathscr{L}}-z_7\hat{J}_7(z_5,z_7)\ .$$

- **Endocytosis:** we make use of Eq. (4) in the Mmin text and Eq. (S18)

$$S_0\delta(x_5)\delta(x_7)\xrightarrow{\mathscr{L}}S_0\ .$$

Now that we have established, term by term, the two-dimensional Laplace transform of the Boltzmann equation, we take a look at the individual mathematical models considered for Rab5 and Rab7 activation/deactivation, the cut-off switch and the toggle-switch models.

Cut-off switch model

We first consider the cut-off switch model and the precise expression of the divergence of the current under the two-dimensional Laplace transform.

- **Rate of change of Rab5:** from the definition of J_5, Eq. (12) in the main text, and Eq. (S10) and Eq. (S11)

$$\begin{aligned} J_5(x_5,x_7) &= (v_{50} - v_{55}x_5 - v_{57}x_7)\, n(x_5,x_7;t) \\ &\xrightarrow{\mathscr{L}} \hat{J}_5(z_5,z_7) = v_{50}\,\hat{n}(z_5,z_7;t) + v_{55}\partial_{z_5}[\,\hat{n}(z_5,z_7;t)] + v_{57}\partial_{z_7}[\,\hat{n}(z_5,z_7;t)]\,. \end{aligned} \tag{S20}$$

- **Rate of change of Rab7:** from the definition of J_7, Eq. (13) in the main text, and Eq. (S10) and Eq. (S11)

$$\begin{aligned} J_7(x_5,x_7) &= (v_{70} + v_{75}x_5 - v_{77}x_7)\, n(x_5,x_7;t) \\ &\xrightarrow{\mathscr{L}} \hat{J}_7(z_5,z_7) = v_{70}\,\hat{n}(z_5,z_7;t) - v_{75}\partial_{z_5}[\,\hat{n}(z_5,z_7;t)] + v_{77}\partial_{z_7}[\,\hat{n}(z_5,z_7;t)]\,. \end{aligned} \tag{S21}$$

We are now ready to bring all the previous results together for the cutt-off switch model. We find the Laplace transform of Eq. (5) in the main text is given by

$$\begin{aligned} \partial_t\,\hat{n}(z_5,z_7;t) = \frac{K_{FUS}^{(0)}}{2}\,[\hat{n}(z_5,z_7;t)]^2 - \frac{K_{FUS}^{(5)}}{2}\partial_{z_5}\,[\hat{n}(z_5,z_7;t)]^2 + \frac{K_{FUS}^{(7)}}{2}\partial_{z_7}\,[\hat{n}(z_5,z_7;t)]^2 - K_{FUS}^{(0)}N(t)\,\hat{n}(z_5,z_7;t) \\ + K_{FUS}^{(5)}\left[N(t)\partial_{z_5}\,\hat{n}(z_5,z_7;t) - R_5(t)\,\hat{n}(z_5,z_7;t)\right] + K_{FUS}^{(7)}\left[-N(t)\partial_{z_7}\,\hat{n}(z_5,z_7;t) + R_7(t)\,\hat{n}(z_5,z_7;t)\right] \\ + \frac{K_{FIS}^{(0)}}{4}\,[\hat{n}(z_5/2,z_7/2;t)]^2 - \frac{K_{FIS}^{(0)}}{8}\,\hat{n}(z_5,z_7;t) \\ - \mu_0\,\hat{n}(z_5,z_7;t) - z_5\left[v_{50}\,\hat{n}(z_5,z_7;t) + v_{55}\partial_{z_5}\,\hat{n}(z_5,z_7;t) + v_{57}\partial_{z_7}\,\hat{n}(z_5,z_7;t)\right] \\ - z_7\left[v_{70}\,\hat{n}(z_5,z_7;t) - v_{75}\partial_{z_5}\,\hat{n}(z_5,z_7;t) + v_{77}\partial_{z_7}\,\hat{n}(z_5,z_7;t)\right] + S_0\,. \end{aligned} \tag{S22}$$

The differential equations for the first order moments, Eqs. (17)-(19), can be derived from Eq. (S22) after one makes the following identifications

$$\begin{aligned} N(t) &= \hat{n}(0,0;t)\,, \\ R_5(t) \equiv \langle x_5\rangle &= -\partial_{z_5}\,\hat{n}(0,0;t)\,, \\ R_7(t) \equiv \langle x_7\rangle &= -\partial_{z_7}\,\hat{n}(0,0;t)\,, \\ \langle x_5^2\rangle &= \partial_{z_5,z_5}\,\hat{n}(0,0;t)\,, \\ \langle x_7^2\rangle &= \partial_{z_7,z_7}\,\hat{n}(0,0;t)\,, \\ \langle x_5x_7\rangle &= \partial_{z_5,z_7}\,\hat{n}(0,0;t)\,, \end{aligned}$$

$$\sigma_5^2 = \langle x_5^2\rangle - \langle x_5\rangle^2 \text{ (variance)}\,, \tag{S23}$$

$$\sigma_7^2 = \langle x_7^2\rangle - \langle x_7\rangle^2 \text{ (variance)}\,, \tag{S24}$$

$$\sigma_{57}^2 = \langle x_5x_7\rangle - \langle x_5\rangle\langle x_7\rangle \text{ (covariance)}\,, \tag{S25}$$

where the derivative has been taken before setting $z_5 = 0 = z_7$.

The equations for the second order moments, variances and covariance, can be computed in the same way as those for the

first order moments, and are given by

$$\begin{aligned}\frac{d\langle x_5^2\rangle}{dt} &= K_{FUS}^{(0)}R_5^2 + 2K_{FUS}^{(5)}R_5\langle x_5^2\rangle - 2K_{FUS}^{(7)}R_5\langle x_5x_7\rangle \\ &- \frac{1}{16}K_{FIS}^{(0)}\langle x_5^2\rangle + 2v_{50}R_5 - 2v_{55}\langle x_5^2\rangle - 2v_{57}\langle x_5x_7\rangle - \mu_0\langle x_5^2\rangle , \end{aligned} \tag{S26}$$

$$\begin{aligned}\frac{d\langle x_7^2\rangle}{dt} &= K_{FUS}^{(0)}R_7^2 + 2K_{FUS}^{(5)}R_7\langle x_5x_7\rangle - 2K_{FUS}^{(7)}R_7\langle x_7^2\rangle \\ &- \frac{1}{16}K_{FIS}^{(0)}\langle x_7^2\rangle + 2v_{70}R_7 + 2v_{75}\langle x_5x_7\rangle - 2v_{77}\langle x_7^2\rangle - \mu_0\langle x_7^2\rangle , \end{aligned} \tag{S27}$$

$$\begin{aligned}\frac{d\langle x_5x_7\rangle}{dt} &= K_{FUS}^{(0)}R_5R_7 + K_{FUS}^{(5)}R_5\langle x_5x_7\rangle + K_{FUS}^{(5)}R_7\langle x_5^2\rangle - K_{FUS}^{(7)}R_5\langle x_7^2\rangle \\ &- K_{FUS}^{(7)}R_7\langle x_5x_7\rangle - \frac{1}{16}K_{FIS}^{(0)}\langle x_5x_7\rangle + v_{70}R_5 + v_{50}R_7 \\ &- v_{55}\langle x_5x_7\rangle - v_{57}\langle x_7^2\rangle + v_{75}\langle x_5^2\rangle - v_{77}\langle x_5x_7\rangle - \mu_0\langle x_5x_7\rangle . \end{aligned} \tag{S28}$$

These three ordinary differential equations, together with equations (S23)-(S25), allow us to obtain the time course of the standard deviations of R_5 and R_7 (and, indirectly, of the endosomal pH).

Toggle-switch model

In the case of the toggle-switch model, the precise expressions for the current are non-linear. We make use of Eq. (14) and Eq. (15) in the main text to write

- **Rate of change of Rab5:** from the definition of J_5, Eq. (14) in the main text, and Eq. (S10) and Eq. (S11)

$$\begin{aligned}J_5(x_5,x_7) &= \left(v_{50} - v_{55}x_5 + \frac{v_{55}}{K_{55}}x_5^2\right)\, n(x_5,x_7;t) \\ &\xrightarrow{\mathscr{L}} \hat{J}_5(z_5,z_7) = v_{50}\,\hat{n}(z_5,z_7;t) + v_{55}\partial_{z_5}[\,\hat{n}(z_5,z_7;t)] + \frac{v_{55}}{K_{55}}\partial_{z_5}^2[\,\hat{n}(z_5,z_7;t)] . \end{aligned} \tag{S29}$$

- **Rate of change of Rab7:** from the definition of J_7, Eq. (15) in the main text, and Eq. (S10) and Eq. (S11)

$$\begin{aligned}J_7(x_5,x_7) &= \left(v_{70} + v_{75}x_5 - \frac{v_{75}}{K_{75}}x_5^2 - v_{77}x_7\right)\, n(x_5,x_7;t) \\ &\rightarrow \hat{J}_7(z_5,z_7) = v_{70}\,\hat{n}(z_5,z_7;t) - v_{75}\partial_{z_5}[\,\hat{n}(z_5,z_7;t)] - \frac{v_{75}}{K_{75}}\partial_{z_5}^2[\,\hat{n}(z_5,z_7;t)] + v_{77}\partial_{z_7}[\,\hat{n}(z_5,z_7;t)] . \end{aligned} \tag{S30}$$

We are now ready to bring all the previous results together for the toggle-switch model. We find the Laplace transform of Eq. (5) in the main text is given by

$$\begin{aligned}\partial_t\,\hat{n}(z_5,z_7;t) &= \frac{K_{FUS}^{(0)}}{2}\,[\hat{n}(z_5,z_7;t)]^2 - \frac{K_{FUS}^{(5)}}{2}\partial_{z_5}\,[\hat{n}(z_5,z_7;t)]^2 + \frac{K_{FUS}^{(7)}}{2}\partial_{z_7}\,[\hat{n}(z_5,z_7;t)]^2 - K_{FUS}^{(0)}N(t)\,\hat{n}(z_5,z_7;t) \\ &+ K_{FUS}^{(5)}\left[N(t)\partial_{z_5}\,\hat{n}(z_5,z_7;t) - R_5(t)\,\hat{n}(z_5,z_7;t)\right] + K_{FUS}^{(7)}\left[-N(t)\partial_{z_7}\,\hat{n}(z_5,z_7;t) + R_7(t)\,\hat{n}(z_5,z_7;t)\right] \\ &+ \frac{K_{FIS}^{(0)}}{4}\,[\hat{n}(z_5/2,z_7/2;t)]^2 - \frac{K_{FIS}^{(0)}}{8}\,\hat{n}(z_5,z_7;t) \\ &- \mu_0\,\hat{n}(z_5,z_7;t) - z_5\left[v_{50}\,\hat{n}(z_5,z_7;t) + v_{55}\partial_{z_5}\,\hat{n}(z_5,z_7;t) + \frac{v_{55}}{K_{55}}\partial_{z_5}^2\,\hat{n}(z_5,z_7;t)\right] \\ &- z_7\left[v_{70}\,\hat{n}(z_5,z_7;t) - v_{75}\partial_{z_5}\,\hat{n}(z_5,z_7;t) - \frac{v_{75}}{K_{75}}\partial_{z_5}^2\,\hat{n}(z_5,z_7;t) + v_{77}\partial_{z_7}\,\hat{n}(z_5,z_7;t)\right] + S_0 . \end{aligned} \tag{S31}$$

The differential equations for the first order moments, Eqs. (17),(21)-(22), can be derived from Eq. (S22) in the same way as for the cut-off switch model. We note that the differential equation for the mean number of endosomes, $N(t)$, is the same for

both models, since the specific form of the currents, $J_5(x_5,x_7)$ and $J_7(x_5,x_7)$, does not change the mean number of endosomes. However, as it should be expected, the differential equations for $R_5(t)$ and $R_7(t)$ depend on the choice of currents. We have

$$\frac{dR_5}{dt} = v_{50}N(t) - v_{55}R_5 + \frac{v_{55}}{K_{55}}\langle x_5^2\rangle - \mu_0 R_5 \,, \tag{S32}$$

$$\frac{dR_7}{dt} = v_{70}N(t) + v_{75}R_5 - \frac{v_{75}}{K_{75}}\langle x_5^2\rangle - v_{77}R_7 - \mu_0 R_7 \,. \tag{S33}$$

We note that the non-linear nature of the currents in this model implies that the differential equations for the first order moments, Eq. (S32) and Eq. (S33), depend on the second order moments. At the level of the Laplace transform, this non-linearity implies that (for $k=5,7$) $\hat{J}_k$ involves second order derivatives of $\hat{n}(z_5,z_7;t)$. Thus, the equations for the first order moments involve the second order ones, and so on. If we define the joint cumulants[49], $\kappa_{i,j,k}$, where $i,j,k=5,7$, as follows:

$$\kappa_i = \langle x_i\rangle \,, \tag{S34}$$

$$\kappa_{i,j} = \langle x_i x_j\rangle - \langle x_i\rangle\langle x_j\rangle \,, \tag{S35}$$

$$\kappa_{i,j,k} = \langle x_i x_j x_k\rangle + 2\langle x_i\rangle\langle x_j\rangle\langle x_k\rangle - \langle x_i x_j\rangle\langle x_k\rangle - \langle x_k x_i\rangle\langle x_j\rangle - \langle x_j x_k\rangle\langle x_i\rangle \,, \tag{S36}$$

then we can, for the sake of simplicity, make use of a zero-cumulant moment-closure approximation[50]. This approximation implies the following choices for the relevant cumulants

$$\begin{cases} \kappa_{5,5}\to 0 & \Rightarrow \langle x_5^2\rangle = \langle x_5\rangle^2 = R_5^2 \,, \\ \kappa_{7,7}\to 0 & \Rightarrow \langle x_7^2\rangle = \langle x_7\rangle^2 = R_7^2 \,, \\ \kappa_{5,7}\to 0 & \Rightarrow \langle x_5x_7\rangle = \langle x_5\rangle\langle x_7\rangle = R_5R_7 \,, \\ \kappa_{5,5,5}\to 0 & \Rightarrow \langle x_5^3\rangle = -2R_5^3 + 3R_5\langle x_5^2\rangle \,, \\ \kappa_{5,5,7}\to 0 & \Rightarrow \langle x_5^2x_7\rangle = -2R_5^2R_7 + R_7\langle x_5^2\rangle + 2R_5\langle x_5x_7\rangle \,. \end{cases}$$

If we make use of the zero-cumulant moment-closure approximation above in Eq. (S32) and Eq. (S33), we obtain Eq. (21) and and Eq. (22), respectively. Similarly, for the second order moments we find the following equations

$$\begin{aligned} \frac{d\langle x_5^2\rangle}{dt} &= K_{FUS}^{(0)}R_5^2 + 2K_{FUS}^{(5)}R_5\langle x_5^2\rangle - 2K_{FUS}^{(7)}R_5\langle x_5x_7\rangle - \frac{1}{16}K_{FIS}^{(0)}\langle x_5^2\rangle \\ &\quad + 2v_{50}R_5 - 2v_{55}\langle x_5^2\rangle - 2\frac{v_{55}}{K_{55}}\langle x_5^3\rangle - \mu_0\langle x_5^2\rangle \,, \end{aligned} \tag{S37}$$

$$\begin{aligned} \frac{d\langle x_7^2\rangle}{dt} &= K_{FUS}^{(0)}R_7^2 + 2K_{FUS}^{(5)}R_7\langle x_5x_7\rangle - 2K_{FUS}^{(7)}R_7\langle x_7^2\rangle - \frac{1}{16}K_{FIS}^{(0)}\langle x_7^2\rangle \\ &\quad + 2v_{70}R_7 + 2v_{75}\langle x_5x_7\rangle + 2\frac{v_{75}}{K_{75}}\langle x_5^2x_7\rangle - 2v_{77}\langle x_7^2\rangle - \mu_0\langle x_7^2\rangle \,, \end{aligned} \tag{S38}$$

$$\begin{aligned} \frac{d\langle x_5x_7\rangle}{dt} &= K_{FUS}^{(0)}R_5R_7 + K_{FUS}^{(5)}\left[R_5\langle x_5x_7\rangle + R_7\langle x_5^2\rangle\right] - K_{FUS}^{(7)}\left[R_5\langle x_7^2\rangle + R_7\langle x_5x_7\rangle\right] - \frac{1}{16}K_{FIS}^{(0)}\langle x_5x_7\rangle \\ &\quad + v_{50}R_7 - v_{55}\langle x_5x_7\rangle - \frac{v_{55}}{K_{55}}\langle x_5^2x_7\rangle \\ &\quad + v_{70}R_5 + v_{75}\langle x_5^2\rangle + \frac{v_{75}}{K_{75}}\langle x_5^3\rangle - v_{77}\langle x_5x_7\rangle - \mu_0\langle x_5x_7\rangle \,. \end{aligned} \tag{S39}$$

If we now make use of the moment-closure approximation, the previous equations can be written as follows:

$$\begin{aligned} \frac{d\langle x_5^2\rangle}{dt} &= K_{FUS}^{(0)}R_5^2 + 2K_{FUS}^{(5)}R_5\langle x_5^2\rangle - 2K_{FUS}^{(7)}R_5\langle x_5x_7\rangle - \frac{1}{16}K_{FIS}^{(0)}\langle x_5^2\rangle \\ &\quad + 2v_{50}R_5 - 2v_{55}\langle x_5^2\rangle - 2\frac{v_{55}}{K_{55}}(-2R_5^3 + 3R_5\langle x_5^2\rangle) - \mu_0\langle x_5^2\rangle \,, \end{aligned} \tag{S40}$$

$$\begin{aligned} \frac{d\langle x_7^2\rangle}{dt} &= K_{FUS}^{(0)}R_7^2 + 2K_{FUS}^{(5)}R_7\langle x_5x_7\rangle - 2K_{FUS}^{(7)}R_7\langle x_7^2\rangle - \frac{1}{16}K_{FIS}^{(0)}\langle x_7^2\rangle \\ &\quad + 2v_{70}R_7 + 2v_{75}\langle x_5x_7\rangle + 2\frac{v_{75}}{K_{75}}(-2R_5^2R_7 + R_7\langle x_5^2\rangle + 2R_5\langle x_5x_7\rangle) - 2v_{77}\langle x_7^2\rangle - \mu_0\langle x_7^2\rangle \,, \end{aligned} \tag{S41}$$

$$\begin{aligned} \frac{d\langle x_5x_7\rangle}{dt} &= K_{FUS}^{(0)}R_5R_7 + K_{FUS}^{(5)}\left[R_5\langle x_5x_7\rangle + R_7\langle x_5^2\rangle\right] - K_{FUS}^{(7)}\left[R_5\langle x_7^2\rangle + R_7\langle x_5x_7\rangle\right] - \frac{1}{16}K_{FIS}^{(0)}\langle x_5x_7\rangle \\ &\quad + v_{50}R_7 - v_{55}\langle x_5x_7\rangle - \frac{v_{55}}{K_{55}}(-2R_5^2R_7 + R_7\langle x_5^2\rangle + 2R_5\langle x_5x_7\rangle) \\ &\quad + v_{70}R_5 + v_{75}\langle x_5^2\rangle + \frac{v_{75}}{K_{75}}(-2R_5^3 + 3R_5\langle x_5^2\rangle) - v_{77}\langle x_5x_7\rangle - \mu_0\langle x_5x_7\rangle \,. \end{aligned} \tag{S42}$$

Section 3: Fitted parameters and sensitivity analysis

We have made use of the software Copasi (Version 4.30.240 for Linux) to perform parameter fitting with three different methods: Levenberg-Marquardt, steepest descent, and Hooke and Jeeves methods[51]. The results converged in all three cases. In Table S1, we summarise the best-fit parameters for the three models introduced in this paper.

Parameter	Cut-off	Reduced	Toggle-switch
$K_{FIS}^{(0)}$	4.54	4.48	0.7723
$K_{FUS}^{(0)}$	0.0032	0.0032	0.0039
v_{50}	1.3×10^{-5}	1.2×10^{-5}	1.3×10^{-5}
$v_{50}N_{ss}$	0.0050	0.0045	0.0050
v_{57}	0.0040	0.0036	—
v_{77}	0.0013	0.0010	0.0013
v_{75}	0.0032	0.0039	0.00314
v_{55}	0.0062	0.0058	0.0062
v_{70}	1.2×10^{-6}	—	1.2×10^{-6}
S_0	0.4389	—	0.1586
μ_0	1.0×10^{-6}	—	1.0×10^{-6}
$K_{FUS}^{(5)}$	2.0×10^{-6}	—	1.8×10^{-6}
$K_{FUS}^{(7)}$	1.2×10^{-5}	—	1.2×10^{-6}
K_{55}	—	—	0.45
K_{55}	—	—	0.53

Table S1. Best-fit parameters obtained by three different methods as implemented in the software Copasi (Version 4.30.240 for Linux)[51] for: i) the cut-off switch model defined by Eqs. (17)-(19) in the main text, ii) the reduced model defined by Eq. (S44) and Eq. (S45), and iii) the toggle-switch model defined by Eq. (17), and Eqs. (21) All parameter values are given in units of seconds^{-1}.

We also used the Copasi built-in (relative) sensitivity analysis algorithm. Tables S2-S5 provide a summary of the results of that algorithm for the three models used: cut-off switch model, toggle-switch model and the model in Ref.[30]. In each table, the column *Aggregated* is the square root of the sum of the squares of each sensitivity. This measure (also computed by Copasi) gives an idea of the most relevant parameters (highest value in that column). Mathematically, we have

$$\text{Aggregated} \equiv \sqrt{\Sigma_N^2 + \Sigma_{R_5}^2 + \Sigma_{R_7}^2} \,. \tag{S43}$$

Consider, for instance, the variable N and the parameter S_0. The value showed in the table corresponds to

$$\frac{S_0}{\Sigma_N}\frac{d\Sigma_N}{dS_0} = 0.0005 \,.$$

That is, every order of magnitude that we increase S_0 only produces an increase of $10^{0.0005} \simeq 1.001$ in the mean number of endosomes (a mere $\sim 0.1\%$ increase). Thus, values close to 1 correspond, roughly, to a linear dependence between variable and parameter and values close to -1, an inverse proportionality. In addition, we have added the column *Normalised*, where we divide the *Aggregated* column by the maximum of all parameters. For instance, in Table S2, the maximum is 1.7294 corresponding to $K_{FUS}^{(0)}$.

From Table S2 we can derive a simpler model, where we drop the less sensitive parameters, namely, S_0, v_{70} $K_{FUS}^{(5)}$ and $K_{FUS}^{(7)}$. We have also set the number of endosomes to a constant, given by the steady state value of the full model. Thus, starting from Eqs. (18)-(19) in the main text, the resulting model is given by the following equations

$$\frac{dR_5}{dt} = v_{50}N_{ss} - (v_{55} + \mu_0)R_5 - v_{57}R_7 \,, \tag{S44}$$

$$\frac{dR_7}{dt} = v_{75}R_5 - (v_{77} + \mu_0)R_7 \,, \tag{S45}$$

with parameters shown in Table S1 and sensitivities in Table S3. Note that, from Table S2, only $K_{FIS}^{(0)}$ and $K_{FUS}^{(0)}$ have strong

Parameter	Σ_N	Σ_{R_5}	Σ_{R_7}	Aggregated	Normalised
$K_{FUS}^{(0)}$	-0.9986	-0.9984	-0.9984	1.7294	1.0000
$K_{FIS}^{(0)}$	0.9990	0.9955	1.0007	1.7293	0.9999
v_{50}	0.0000	1.4462	0.8411	1.6731	0.9674
v_{57}	0.0000	-1.1510	-0.6141	1.3046	0.7543
v_{77}	0.0000	1.0718	-0.3886	1.1401	0.6593
v_{75}	0.0000	-0.7050	0.2265	0.7405	0.4282
v_{55}	0.0000	-0.4462	0.1589	0.4737	0.2739
v_{70}	0.0000	-0.2259	-0.2519	0.3384	0.1957
S_0	0.0005	0.0005	0.0005	0.0009	0.0005
μ_0	-0.0001	0.0007	-0.0003	0.0008	0.0004
$K_{FUS}^{(5)}$	$< 10^{-4}$	$< 10^{-4}$	$< 10^{-4}$	$< 10^{-4}$	$< 10^{-4}$
$K_{FUS}^{(7)}$	$< 10^{-4}$	$< 10^{-4}$	$< 10^{-4}$	$< 10^{-4}$	$< 10^{-4}$

Table S2. Sensitivity analysis of the mathematical model described by Eqs. (17)-(19), corresponding to the cut-off switch hypothesis in Fig. 1 in the main text, as computed by three different methods implemented in the software Copasi (Version 4.30.240 for Linux)[51]. The column *Aggregated* is defined in Eq. (S43). The column *Normalised* is the value in *Aggregated* divided by the maximum value in that column. The thin dotted lines are a guide to the eye to separate the most sensitive parameters (top part of the table) and the least (bottom part).

sensitivities on the variable N. We can then safely assume that

$$N_{ss} = \frac{K_{FIS}^{(0)}}{4K_{FUS}^{(0)}} .$$

This is confirmed in Fig. S1, where the number of endosomes quickly converges to its steady state.

Parameter	Σ_N	Σ_{R_5}	Σ_{R_7}	Aggregated	Normalised
$K_{FIS}^{(0)}$	1.0000	0.9995	1.0001	1.7318	1.0000
$K_{FUS}^{(0)}$	-0.9990	-0.9992	-0.9991	1.7305	0.9992
v_{50}	$< 10^{-4}$	0.9991	1.0013	1.4145	0.8168
v_{57}	$< 10^{-4}$	-0.7805	-0.7288	1.0679	0.6166
v_{75}	$< 10^{-4}$	-0.7805	0.2704	0.8260	0.4770
v_{77}	$< 10^{-4}$	0.7556	-0.2928	0.8103	0.4679
v_{55}	$< 10^{-4}$	-0.1181	-0.3022	0.3245	0.1874

Table S3. Sensitivity analysis of the reduced model computed by three different methods implemented in the software Copasi (Version 4.30.240 for Linux)[51]. The column *Aggregated* is defined in Eq. S43. The column *Normalised* is the value in *Aggregated* divided by the maximum value in that column.

Finally, and for the sake of completeness, in Table S4 we summarise the sensitivity analysis for the toggle-switch model.

Section 4: Study of two previous models of Rab dynamics

Mathematical model in Ref.[21]

The authors in Ref.[21] encoded the dynamics of Fig. 1 in the main text as a set of four ODEs, where each reaction term was fitted to different mathematical equations. One set of functions, as documented in http://biomodels.caltech.edu/BIOMD0000000174. provides the following equations (with t the variable describing experimental time)

$$\frac{\mathrm{d}[\mathrm{Rab5-GDP}]}{\mathrm{d}t} = v_5 - \frac{[\mathrm{Rab5-GDP}] \cdot \frac{k_{e,5} \cdot t}{T_0 + t}}{1 + e^{k_{g,5} - [\mathrm{Rab5-GTP}] \cdot k_{f,5}}} - k_5 \cdot [\mathrm{Rab5-GDP}] + \frac{k_{e,57} \cdot [\mathrm{Rab5-GTP}]}{1 + e^{k_{g,75} - [\mathrm{Rab7-GTP}] \cdot k_{f,57}}} + k_7 \cdot [\mathrm{Rab5-GTP}] , \quad \text{(S46)}$$

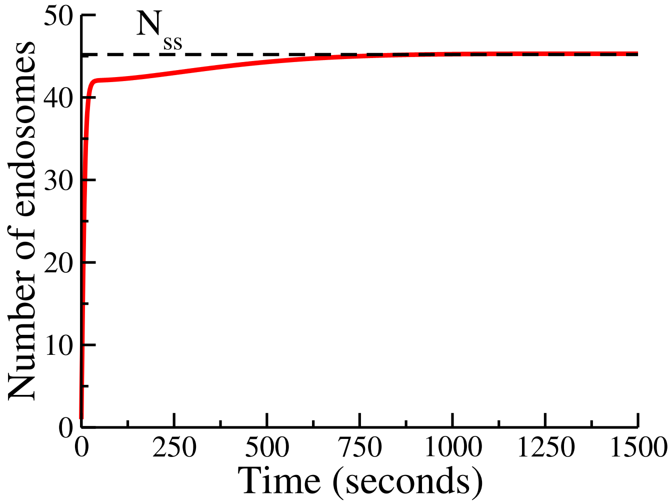

Figure S1. Time course of the number of endosomes, $N(t)$, for the cut-off switch model (solid red line) and its steady state value, N_{ss}, (black dashed lines). Note that $N(t)$ grows rapidly in the first 40 seconds and reaches the steady state value of the reduced model after 500 seconds. The numerical value is extremely close to the value given by Eq. (23) in the main text.

Parameter	Σ_N	Σ_{R_5}	Σ_{R_7}	Aggregated	Normalised
$K_{FIS}^{(0)}$	0.9990	0.5301	0.8049	1.3881	1.0000
$K_{FUS}^{(0)}$	-0.9985	-0.5301	-0.8029	1.3866	0.9989
v_{70}	$< 10^{-4}$	$< 10^{-4}$	0.6682	0.6682	0.4814
v_{77}	$< 10^{-4}$	$< 10^{-4}$	-0.6632	0.6632	0.4778
v_{50}	$< 10^{-4}$	0.5307	0.1359	0.5478	0.3947
K_{55}	$< 10^{-4}$	-0.4688	-0.1193	0.4838	0.3485
v_{75}	$< 10^{-4}$	$< 10^{-4}$	0.3318	0.3318	0.2390
K_{75}	$< 10^{-4}$	$< 10^{-4}$	0.0764	0.0764	0.0550
v_{54}	$< 10^{-4}$	-0.0616	-0.0157	0.0636	0.0458
S_0	0.0005	0.0003	0.0007	0.0009	0.0007
μ_0	$< 10^{-4}$	$< 10^{-4}$	-0.0006	0.0005	0.0004
$K_{FUS}^{(7)}$	$< 10^{-4}$	$< 10^{-4}$	$< 10^{-4}$	$< 10^{-4}$	$< 10^{-4}$
$K_{FUS}^{(5)}$	$< 10^{-4}$	$< 10^{-4}$	$< 10^{-4}$	$< 10^{-4}$	$< 10^{-4}$

Table S4. Sensitivity analysis for the toggle-switch model computed by three different methods implemented in the software Copasi (Version 4.30.240 for Linux)[51]. The column *Aggregated* is defined in Eq. S43. The column *Normalised* is the value in *Aggregated* divided by the maximum value in that column. The thin dotted lines are a guide to the eye to separate the most sensitive parameters (top part of the table) and the least (bottom part).

Parameter	$\Sigma_{Rab5-GDP}$	$\Sigma_{Rab5-GTP}$	$\Sigma_{Rab7-GDP}$	$\Sigma_{Rab7-GTP}$	Aggregated	Normalised
k_7	0.0022	28.8007	-1.0044	-7.8522	29.8688	1.0000
$k_{1,7}$	0.0021	28.7917	-0.0055	-7.8498	29.8426	0.9991
v_7	-0.0018	-27.1203	1.0046	7.57364	28.17588	0.9433
$k_{e,7}$	-0.0018	-26.6033	0.0045	7.4276	27.6207	0.9247
$k_{g,7}$	0.0007	9.4375	-0.0020	-2.5938	9.7875	0.3277
$k_{g,57}$	0.0004	4.3526	-0.0010	-1.1988	4.5147	0.1512
$k_{f,57}$	0.0003	3.3470	-0.0007	-0.9223	3.4718	0.1162
$k_{g,75}$	-0.0005	0.2003	0.0012	1.6523	1.6643	0.0557
h_7	0.0003	1.2028	-0.0007	-0.3313	1.2476	0.0418
k_5	0.9998	0.0216	0.0002	0.2905	1.0414	0.0349
v_5	-0.9989	-0.0135	-0.0003	-0.2928	1.0410	0.0349
$k_{f,75}$	-0.0002	0.0541	0.0006	0.6927	0.6948	0.0233
$k_{e,57}$	0.0001	-0.5757	0.0001	0.1589	0.5972	0.0200
$k_{e,5}$	-0.0002	0.0205	0.0002	0.2908	0.2915	0.0098
$k_{e,75}$	0.0001	-0.0133	-0.0002	-0.2268	0.2272	0.0076
$k_{g,5}$	$< 10^{-4}$	-0.0074	-0.0001	-0.1559	0.1561	0.0052
$k_{1,5}$	$< 10^{-4}$	-0.0068	-0.0001	-0.1354	0.1355	0.0045
$k_{f,5}$	$< 10^{-4}$	-0.0007	$< 10^{-4}$	-0.0661	0.0661	0.0022
T_0	$< 10^{-4}$	0.0053	$< 10^{-4}$	-0.0390	0.03938	0.0013

Table S5. Sensitivity analysis of the model in Ref.[21] (corresponding to Eqs. (S46)-(S49) of Section 2 in Supporting information) obtained by three different methods implemented in the software Copasi (Version 4.30.240 for Linux)[51]. The column *Aggregated* is defined in Eq. S43. The column *Normalised* is the value in *Aggregated* divided by the maximum value in that column. The thin dotted lines are a guide to the eye to separate the most sensitive parameters (top part of the table) and the least (bottom part).

$$\frac{d[\mathrm{Rab5-GTP}]}{dt} = \frac{[\mathrm{Rab5-GDP}]\cdot\frac{k_{e,5}\cdot t}{T_0+t}}{1+e^{k_{g,5}-[\mathrm{Rab5-GTP}]\cdot k_{f,5}}} - \frac{k_{e,57}\cdot[\mathrm{Rab5-GTP}]}{1+e^{k_{g,57}-[\mathrm{Rab7-GTP}]\cdot k_{f,57}}} - k_5\cdot[\mathrm{Rab5-GTP}]\,, \tag{S47}$$

$$\frac{d[\mathrm{Rab7-GDP}]}{dt} = v_7 - \frac{[\mathrm{Rab7-GDP}]\cdot k_{e,7}\cdot[\mathrm{Rab7-GTP}]^{h_7}}{k_{g,7}+[\mathrm{Rab7-GTP}]^{h_7}} - \frac{k_{e,57}\cdot[\mathrm{Rab7-GDP}]}{1+e^{k_{g,57}-[\mathrm{Rab5-GTP}]\cdot k_{f,57}}} - k_7\cdot[\mathrm{Rab7-GDP}] + k_{1,7}\cdot[\mathrm{Rab7-GTP}]\,, \tag{S48}$$

$$\frac{d[\mathrm{Rab7-GTP}]}{dt} = \frac{[\mathrm{Rab7-GDP}]\cdot k_{e,7}\cdot[\mathrm{Rab7-GTP}]^{h_7}}{k_{g,7}+[\mathrm{Rab7-GTP}]^{h_7}} + \frac{k_{e,5}\cdot[\mathrm{Rab7-GDP}]}{1+e^{k_{g,5}-[\mathrm{Rab5-GTP}]\cdot k_{f,5}}} - k_{1,7}\cdot[\mathrm{Rab7-GTP}]\,. \tag{S49}$$

Although this model looks clearly more complex than our model (see Eqs. (18)-(19)), numerical integration of these equations (as shown in Fig. 1B in the main text) shows that the variables [Rab5-GDP] and [Rab7-GDP] are almost constant (not shown). Hence, Eqs. (S46)-(S49) are identical to Eqs. (18)-(19) in the main text, after linearisation of the equations for [Rab5-GTP] and [Rab7-GTP], with the following approximations:

$$\frac{[\mathrm{Rab5-GDP}]\cdot\frac{k_{e,5}\cdot t}{T_0+t}}{1+e^{k_{g,5}-[\mathrm{Rab5-GTP}]\cdot k_{f,5}}} \to v_{50}N\,,$$

and

$$\frac{k_{e,5}\cdot[\mathrm{Rab7-GDP}]}{1+e^{k_{g,5}-[\mathrm{Rab5-GTP}]\cdot k_{f,5}}} \to v_{70}N\,.$$

Mathematical model in Ref.[30]

For completeness, we reproduce here the system of equations in Ref.[30]. Note that the first two terms in Eq. (17) in the main text are equivalent to those in Eq. (S53).

$$\frac{d\mathrm{Rab5}_{ee}}{dt} = -\mathrm{Rab5}_{ee}\frac{dN}{dt}\frac{1}{N} - k_{\mathrm{GAP}}(\mathrm{Rab5}_{ee})\mathrm{Rab5}_{ee} + k_{\mathrm{GEF}}(\mathrm{Rab5}_{ee})\mathrm{rab5}_{ee} \;, \tag{S50}$$

$$\frac{d\mathrm{rab5}_{ee}}{dt} = -\mathrm{rab5}_{ee}\frac{dN}{dt}\frac{1}{N} + k_{\mathrm{GAP}}(\mathrm{Rab5}_{ee})\mathrm{Rab5}_{ee} - k_{\mathrm{GEF}}(\mathrm{Rab5}_{ee})\mathrm{rab5}_{ee} + k_1\mathrm{rab5}_{cyt} - k_{-1}\mathrm{rab5}_{ee} \;, \tag{S51}$$

$$\frac{d\mathrm{rab5}_{cyt}}{dt} = -k_1\mathrm{rab5}_{cyt} + k_{-1}\mathrm{rab5}_{ee} \;, \tag{S52}$$

$$\frac{dN}{dt} = -k_{\mathrm{fus}}(\mathrm{Rab5}_{ee})N^2 + k_{\mathrm{fis}}(\mathrm{Rab5}_{ee})N \;. \tag{S53}$$

Note that, as according to our model, the number of endosomes reaches quickly a steady state, the terms proportional to $\frac{dN}{dt}$ are negligible in comparison to the other terms. Similarly, as shown in the Supporting information in Ref.[21], the inactive $rab5_{ee}$ is also almost constant. Thus, we can reduce the equations above to the following system

$$\frac{d\mathrm{Rab5}_{ee}}{dt} = -k_{\mathrm{GAP}}(\mathrm{Rab5}_{ee})\mathrm{Rab5}_{ee} + k_{\mathrm{GEF}}(\mathrm{Rab5}_{ee})\mathrm{rab5}_{ee} \;,$$

$$\frac{dN}{dt} = -k_{\mathrm{fus}}(\mathrm{Rab5}_{ee})N^2 + k_{\mathrm{fis}}(\mathrm{Rab5}_{ee})N \;, \tag{S54}$$

where our notation $k_{\mathrm{GAP}}(\mathrm{Rab5}_{ee}), k_{\mathrm{GEF}}(\mathrm{Rab5}_{ee}), k_{\mathrm{fus}}(\mathrm{Rab5}_{ee})$ and $k_{\mathrm{fis}}(\mathrm{Rab5}_{ee})$ implies that these rates are functions of the variable $\mathrm{Rab5}_{ee}$. Our analysis has shown that the source term, proportional to S_0, and the death term, proportional to μ_0, are only important in the initial and transient regime. So Eq. (17) and Eq. (18) in the main text can be written as:

$$\frac{dR_5}{dt} = v_{50}N - (v_{55} + \mu_0)R_5 - v_{57}R_7 \;,$$

$$\frac{dN}{dt} = -\frac{1}{2}K^{(0)}_{FUS}N^2 + \left(\frac{1}{8}K^{(0)}_{FIS} - K^{(5)}_{FUS}R_5 + K^{(7)}_{FUS}R_7\right)N \;, \tag{S55}$$

which are analogous to Eqs. (S54). In our case, the inclusion of the variable R_7 (not included in Ref.[30]) can explain the need to choose more sophisticated mathematical functions for k_{GEF} and k_{GAP} in the equations above. To illustrate this point, in Fig. S2 we show Rab5 as a function of Rab7 for both the experimental data (circles) and the fitted model (solid line). This allows us to conclude that Rab5 depends non-linearly on Rab7. If we describe Rab5, without reference to the dynamics of Rab7, as done in Ref.[30], one would require an equation for R_5 containing non-linearities. In particular, if we denote by $k_{GAP}(Rab5_{ee})$, the coefficient of $Rab5_{ee}$ and by $k_{GEF}(Rab5_{ee})$, the coefficient of $rab5_{ee}$, one can see that the dependence on R_7 in the first case and on N on the second one, are equivalent to the non-linear functions of R_5 in Eq. (S54).

Finally, and as shown in Figures S1a-S1b from Ref.[21], the best-fit for the fusion rate is almost independent of Rab5 (note the large bars in logarithmic scale), while the best-fit for the fission rate consistently changes as a function Rab5. In our framework, this dependence is encapsulated in the factor $K^{(0)}_{FIS}/8 - \mu_0 - K^{(5)}_{FUS} + K^{(7)}_{FUS}$ multiplying the variable N in Eq. (17) in the main text, and the constant coefficient $K^{(0)}_{FUS}$ multiplying N^2. So the we can identify

$$k_{\mathrm{fis}}(\mathrm{Rab5}_{ee}) \rightarrow K^{(0)}_{FIS}/8 - \mu_0 - K^{(5)}_{FUS}R_5 + K^{(7)}_{FUS}R_7 \;,$$

in Eq. S54. This identification clearly shows that $k_{\mathrm{fis}}(\mathrm{Rab5}_{ee})$ is a non-linear function of R_5 since $R_7 = R_7(R_5)$ (see Fig. S2). This completes the connection between the mathematical framework presented here and previous mathematical models of endosome maturation.

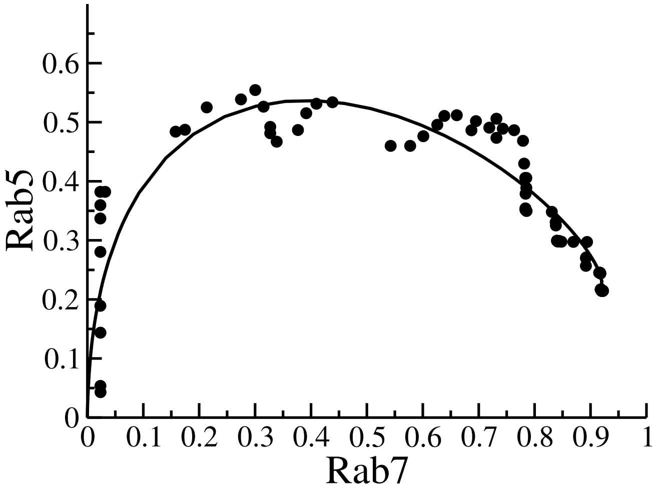

Figure S2. Phase-diagram of Rab5 and Rab7. Circles: experimental data. Solid line: fit to Eqs. (18)-(19) in the main text. This shows that R_5 can be expressed as a non-linear function of R_7. Thus, linear terms of R_5 and R_7 can be misidentified with non-linear functions of R_5 alone.

References

1. Naslavsky, N. & Caplan, S. The enigmatic endosome–sorting the ins and outs of endocytic trafficking. Journal of cell science **131**, jcs216499 (2018).
2. Tokarev, A. A., Alfonso, A. & Segev, N. Overview of intracellular compartments and trafficking pathways. In Trafficking Inside Cells, 3–14 (Springer, 2009).
3. Pfeffer, S. R. Rab GTPase regulation of membrane identity. Current opinion in cell biology **25**, 414–419 (2013).
4. Staring, J., Raaben, M. & Brummelkamp, T. R. Viral escape from endosomes and host detection at a glance. J. cell science **131** (2018).
5. Rink, J., Ghigo, E., Kalaidzidis, Y. & Zerial, M. Rab conversion as a mechanism of progression from early to late endosomes. Cell **122**, 735–749 (2005).
6. Duclos, S. et al. Rab5 regulates the kiss and run fusion between phagosomes and endosomes and the acquisition of phagosome leishmanicidal properties in RAW 264.7 macrophages. Journal of cell science **113**, 3531–3541 (2000).
7. Huotari, J. & Helenius, A. Endosome maturation. The EMBO journal **30**, 3481–3500 (2011).
8. Flipse, J., Wilschut, J. & Smit, J. M. Molecular Mechanisms Involved in Antibody-Dependent Enhancement of Dengue Virus Infection in Humans. Traffic **14**, 25–35 (2013).
9. Krishnan, M. N. et al. Rab 5 is required for the cellular entry of dengue and West Nile viruses. Journal of virology **81**, 4881–4885 (2007).
10. van der Schaar, H. M. et al. Dissecting the cell entry pathway of dengue virus by single-particle tracking in living cells. PLoS pathogens **4**, e1000244 (2008).
11. Chao, L. H., Klein, D. E., Schmidt, A. G., Peña, J. M. & Harrison, S. C. Sequential conformational rearrangements in flavivirus membrane fusion. Elife **3** (2014).
12. van Duijl-Richter, M. K., Blijleven, J. S., van Oijen, A. M. & Smit, J. M. Chikungunya virus fusion properties elucidated by single-particle and bulk approaches. Journal of General Virology **96**, 2122–2132 (2015).
13. Hoornweg, T. E. et al. Dynamics of chikungunya virus cell entry unraveled by single-virus tracking in living cells. Journal of virology **90**, 4745–4756 (2016).
14. Feng, Y., Press, B. & Wandinger-Ness, A. Rab 7: an important regulator of late endocytic membrane traffic. The Journal of cell biology **131**, 1435–1452 (1995).
15. Mellman, I., Fuchs, R. & Helenius, A. Acidification of the endocytic and exocytic pathways. Annual review of biochemistry **55**, 663–700 (1986).
16. Lagache, T., Sieben, C., Meyer, T., Herrmann, A. & Holcman, D. Stochastic acidification, activation of hemagglutinin and escape of influenza viruses from an endosome. Frontiers in Physics **5**, 25 (2017).
17. Binder, B., Goede, A., Berndt, N. & Holzhütter, H.-G. A conceptual mathematical model of the dynamic self-organisation of distinct cellular organelles. PloS One **4**, e8295 (2009).
18. Rodenhuis-Zybert, I. A., Wilschut, J. & Smit, J. M. Dengue virus life cycle: viral and host factors modulating infectivity. Cellular and molecular life sciences **67**, 2773–2786 (2010).
19. Floyd, D. L., Ragains, J. R., Skehel, J. J., Harrison, S. C. & van Oijen, A. M. Single-particle kinetics of influenza virus membrane fusion. Proceedings of the National Academy of Sciences **105**, 15382–15387 (2008).
20. Ivanovic, T., Choi, J. L., Whelan, S. P., van Oijen, A. M. & Harrison, S. C. Influenza-virus membrane fusion by cooperative fold-back of stochastically induced hemagglutinin intermediates. Elife **2** (2013).
21. Del Conte-Zerial, P. et al. Membrane identity and GTPase cascades regulated by toggle and cut-out switches. Molecular systems biology **4**, 206 (2008).
22. Gautreau, A., Oguievetskaia, K. & Ungermann, C. Function and regulation of the endosomal fusion and fission machineries. Cold Spring Harbor perspectives in biology **6**, a016832 (2014).
23. Wandinger-Ness, A. & Zerial, M. Rab proteins and the compartmentalization of the endosomal system. Cold Spring Harbor perspectives in biology **6**, a022616 (2014).
24. Vetter, I. R. & Wittinghofer, A. The guanine nucleotide-binding switch in three dimensions. Science **294**, 1299–1304 (2001).

25. Tashkova, K., Korošec, P., Šilc, J., Todorovski, L. & Džeroski, S. Parameter estimation with bio-inspired meta-heuristic optimization: modeling the dynamics of endocytosis. BMC systems biology **5**, 159 (2011).

26. Vonderheit, A. & Helenius, A. Rab7 associates with early endosomes to mediate sorting and transport of Semliki forest virus to late endosomes. PLoS biology **3**, e233 (2005).

27. Binder, B. & Holzhütter, H.-G. A hypothetical model of cargo-selective rab recruitment during organelle maturation. Cell biochemistry and biophysics **63**, 59–71 (2012).

28. Foret, L. et al. A general theoretical framework to infer endosomal network dynamics from quantitative image analysis. Current Biology **22**, 1381–1390 (2012).

29. Grabe, M. & Oster, G. Regulation of organelle acidity. The J. general physiology **117**, 329–344 (2001).

30. Zeigerer, A. et al. Rab5 is necessary for the biogenesis of the endolysosomal system in vivo. Nature **485**, 465–470 (2012).

31. Castro, M., Lythe, G. & Molina-París, C. The T cells in an ageing virtual mouse. In Stochastic Processes, Multiscale Modeling, and Numerical Methods for Computational Cellular Biology, 127–140 (Springer, 2017).

32. Zerial, M. & McBride, H. Rab proteins as membrane organizers. Nature reviews Molecular cell biology **2**, 107–117 (2001).

33. Smoluchowski, M. v. Brownsche Molekularbewegung und Koagulation von Kolloidteilchen. Phys. Zeits. **17**, 585–599 (1916).

34. Meakin, P. Aggregation kinetics. Physica Scripta **46**, 295 (1992).

35. Lade, S. J., Coelho, M., Tolić, I. M. & Gross, T. Fusion leads to effective segregation of damage during cell division: An analytical treatment. Journal of theoretical biology **378**, 47–55 (2015).

36. Donovan, G. M. & Lythe, G. T cell and reticular network co-dependence in HIV infection. Journal of theoretical biology **395**, 211–220 (2016).

37. Cuesta, J. A., Delius, G. W. & Law, R. Sheldon spectrum and the plankton paradox: two sides of the same coin—a trait-based plankton size-spectrum model. Journal of mathematical biology **76**, 67–96 (2018).

38. Kalaidzidis, Y. et al. Learning microscopic kinetic characteristic of endosomal network by quantitative analysis of snap-shot microscopy images. In Proceedings of the International Conference on Bioinformatics & Computational Biology (BIOCOMP), 1 (The Steering Committee of The World Congress in Computer Science, Computer Engineering and Applied Computing (WorldComp), 2013).

39. Ganusov, V. V. Strong Inference in Mathematical Modeling: A Method for Robust Science in the Twenty-First Century. Frontiers in microbiology **7**, 1131 (2016).

40. Modi, S. et al. A DNA nanomachine that maps spatial and temporal pH changes inside living cells. Nature nanotechnology **4**, 325 (2009).

41. Padilla-Parra, S. et al. Quantitative imaging of endosome acidification and single retrovirus fusion with distinct pools of early endosomes. Proceedings of the National Academy of Sciences **109**, 17627–17632 (2012).

42. Camacho, J. Scaling in steady-state aggregation with injection. Physical Review E **63**, 046112 (2001).

43. Murray, D. H. et al. An endosomal tether undergoes an entropic collapse to bring vesicles together. Nature **537**, 107 (2016).

44. Brett, C. L., Tukaye, D. N., Mukherjee, S. & Rao, R. The yeast endosomal Na+ (K+)/H+ exchanger Nhx1 regulates cellular pH to control vesicle trafficking. Mol. biology cell **16**, 1396–1405 (2005).

45. Foret, L. & Sens, P. Kinetic regulation of coated vesicle secretion. Proceedings of the National Academy of Sciences **105**, 14763–14768 (2008).

46. Lakadamyali, M., Rust, M. J. & Zhuang, X. Ligands for clathrin-mediated endocytosis are differentially sorted into distinct populations of early endosomes. Cell **124**, 997–1009 (2006).

47. Farmer, T., Naslavsky, N. & Caplan, S. Tying trafficking to fusion and fission at the mighty mitochondria. Traffic **19**, 569–577 (2018).

48. Schiff, J. L. The Laplace transform: theory and applications (Springer Science & Business Media, 2013).

49. Daley, D. J. & Vere-Jones, D. An introduction to the theory of point processes: volume II: general theory and structure (Springer Science & Business Media, 2007).

50. Singh, A. & Hespanha, J. P. Approximate moment dynamics for chemically reacting systems. IEEE Transactions on Automatic Control **56**, 414–418 (2010).

51. Hoops, S. et al. COPASI—a complex pathway simulator. Bioinformatics **22**, 3067–3074 (2006). In this work we have used Version 4.30.240 for Linux (`http://copasi.org/Download/`)
